# Anti-CD81 antibodies reduce migration of activated T lymphocytes and attenuate mouse experimental colitis

**DOI:** 10.1038/s41598-020-64012-5

**Published:** 2020-04-24

**Authors:** Takuya Hasezaki, Tadahiko Yoshima, Yukiko Mine

**Affiliations:** 1 External Innovation, Sumitomo Dainippon Pharma Co., Ltd, Osaka, 554-0022 Japan; 2 Applied Bioscience Group, Bioscience Research Laboratory, Sumitomo Chemical Co., Ltd, Osaka, 554-0022 Japan; 3 Group 2, Platform Technology Research Unit, Sumitomo Dainippon Pharma Co., Ltd, Osaka, 554-0022 Japan

**Keywords:** Target identification, Target validation, Inflammatory bowel disease

## Abstract

Inflammatory bowel disease (IBD) is an immunological disease associated with CD4^+^ T cell activation in the intestines. CD81 is a regulator of the immune system with multiple biological functions. Therefore, in this study, we assessed the contribution of CD81 to IBD pathophysiology and the therapeutic efficacy of anti-CD81 antibodies. Expression of CD81 was increased on activated T cells *in vitro* and in colitic mice *in vivo*. Therapeutic effects of anti-CD81 antibodies on colitic symptoms and inflammation were evaluated in mice with colitis, including long-term effects of the antibodies. Treatment with anti-CD81 antibodies improved colitis scores, reduced colon shortening, decreased loss of body weight, and resulted in fewer pathological changes of the colon in colitic mice. Moreover, the increased inflammatory markers in the blood of colitic mice were decreased by anti-CD81 antibodies. The anti-CD81 antibody treatment had long-lasting therapeutic effects on colitic mice, even after cessation of treatment. Two different clones of the anti-mouse CD81 antibody were also effective in mice with colitis. Furthermore, anti-CD81 antibodies reduced migration of CD4^+^ T cells both in colitic mice and *in vitro*. Thus, CD81 contributes to IBD pathology and treatment with anti-CD81 antibodies may be a potential novel therapy for IBD patients.

## Introduction

Inflammatory bowel disease (IBD), including Crohn’s disease and ulcerative colitis, is a group of diseases with chronic and relapsing intestinal inflammation. Current therapies focus on controlling inflammation using immunosuppressants, steroids, or biopharmaceuticals against proinflammatory cytokines and lymphocytes. However, the clinical benefits of current medical treatments are limited and many patients live long-term with the disease after onset at a young age^1,2^. IBD is an immunological disease associated with activation of CD4^+^ T cells in the intestines^3,4^ and the expression of multiple proinflammatory chemokines, including C-X-C chemokine receptor type 4 (CXCR4), which attract leukocytes into inflamed intestines in both IBD mouse models^1^ and patients^2^.

CD81 is a cell surface protein belonging to the tetraspanin superfamily. It has been identified as a component of the B lymphocyte receptor and a host entry factor for the hepatitis C virus^5^. Tetraspanins increase the formation and stability of biologically functional receptors consisting of tetraspanins and other molecules^3^. CD81 associates with various immune molecules on T and B lymphocytes as well as other cell types to facilitate cell-to-cell communication at the immune synapse interface between antigen-presenting cells (APCs) and T lymphocytes^6^. We examined the contribution of CD81 to the pathology of IBD using anti-mouse CD81 antibodies and 2,4,6-trinitrobenzenesulfonic acid (TNBS)-induced colitis to determine its therapeutic potential for IBD. Targeting cell migration is considered as one of the most promising therapeutic approaches for IBD, because mice with TNBS-induced colitis have inflamed colons in which activated CD4^+^ T cells accumulate^7^. The present study aimed to determine the role of CD81 in the pathophysiology of IBD and the therapeutic potential of anti-CD81 antibodies for IBD.

## Results

### CD81 is increased on activated T cells and in mice with TNBS-induced colitis

**Figure 1:**
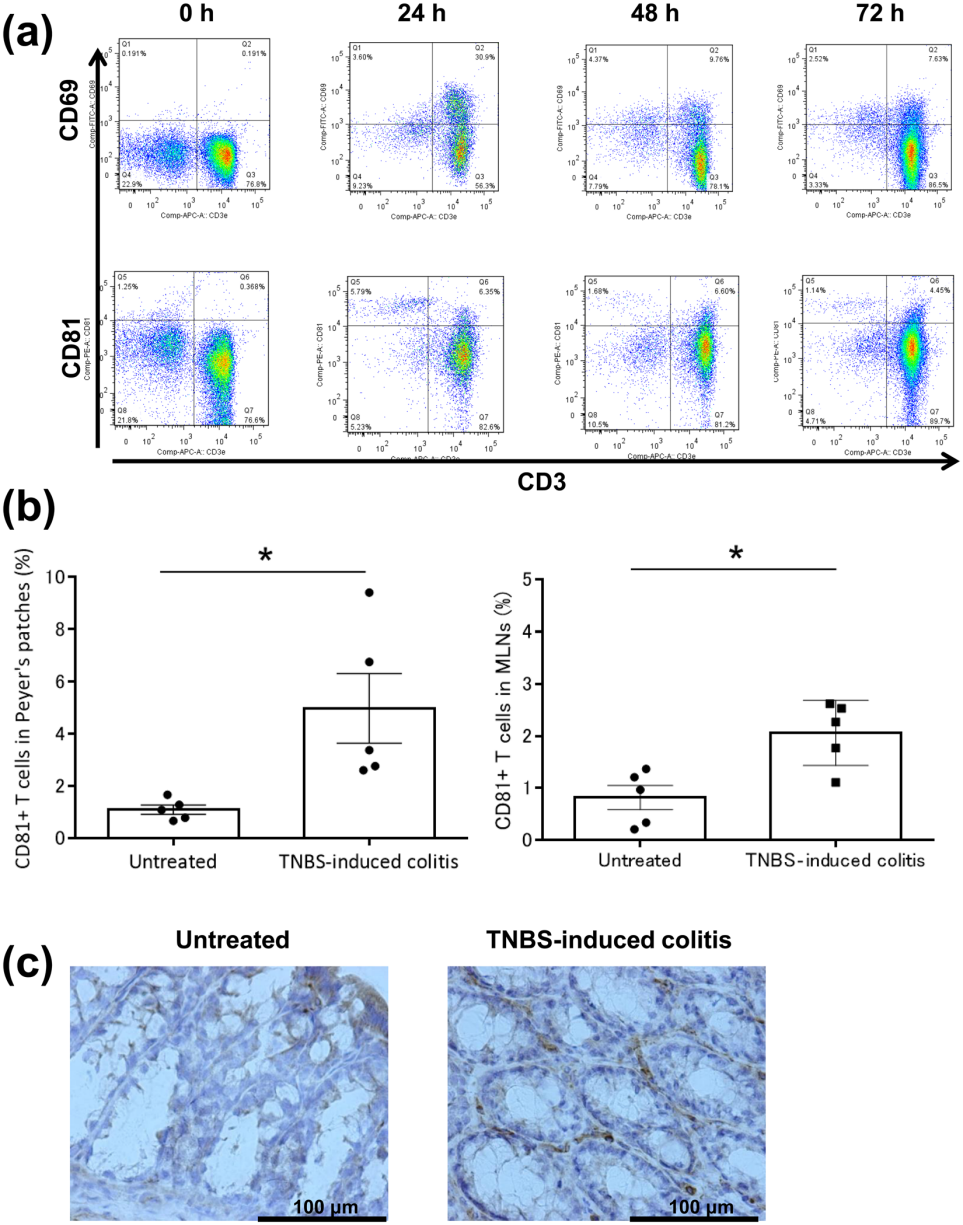
Expression of CD81 in mouse PBMCs stimulated with PHA and IL-2, and in mice with TNBS-induced colitis. (**a**) PBMCs were collected from SJL/J mice and stimulated with PHA and human IL-2 for 0, 24, 48, and 72 h. PBMCs were stained with anti-CD69, anti-CD81 (clone Eat2), and anti-CD3ε (n = 3 per group) antibodies. Then, cell surface markers were analysed using a FACSCanto II. (**b**) Cells in mesenteric lymph nodes (MLNs) from mice with TNBS-induced colitis and untreated mice were stained with anti-CD3e and anti-CD81 antibodies, and then analysed using the FACSCanto II (n = 5 per group). Statistical significance was determined using the Student’s t-test (*p < 0.05). Data are representative of three independent experiments. (**c**) Representative immunohistochemical staining of colons from mice with TNBS-induced colitis and untreated mice. Colons were removed on day 4, fixed with paraformaldehyde, embedded in paraffin, and sectioned. Immunostaining was performed with biotin-labelled hamster IgG or the anti-CD81 antibody.

To examine CD81 expression on activated T cells, peripheral blood mononuclear cells (PBMCs) from SJL/J mice were cultured with phytohemagglutinin (PHA) and IL-2 for 0, 24, 48, and 72 h. CD69 on T cells was maximally increased at 24 h after stimulation, while CD81 was increased from 24 to 72 h (Fig. 1A). CD81^+^ T cells among lymphocytes of the Peyer’s patches and mesenteric lymph nodes of mice with TNBS-induced colitis were increased compared with those of untreated mice (Fig. 1B). Moreover, overall, CD81^+^ cells in the colons of mice with TNBS-induced colitis were increased compared with those of untreated mice (Fig. 1C). Thus, CD81 was increased on activated T cells in mice with colitis.

### Anti-CD81 antibody has short-term effects on TNBS-induced colitic mice

**Figure 2:**
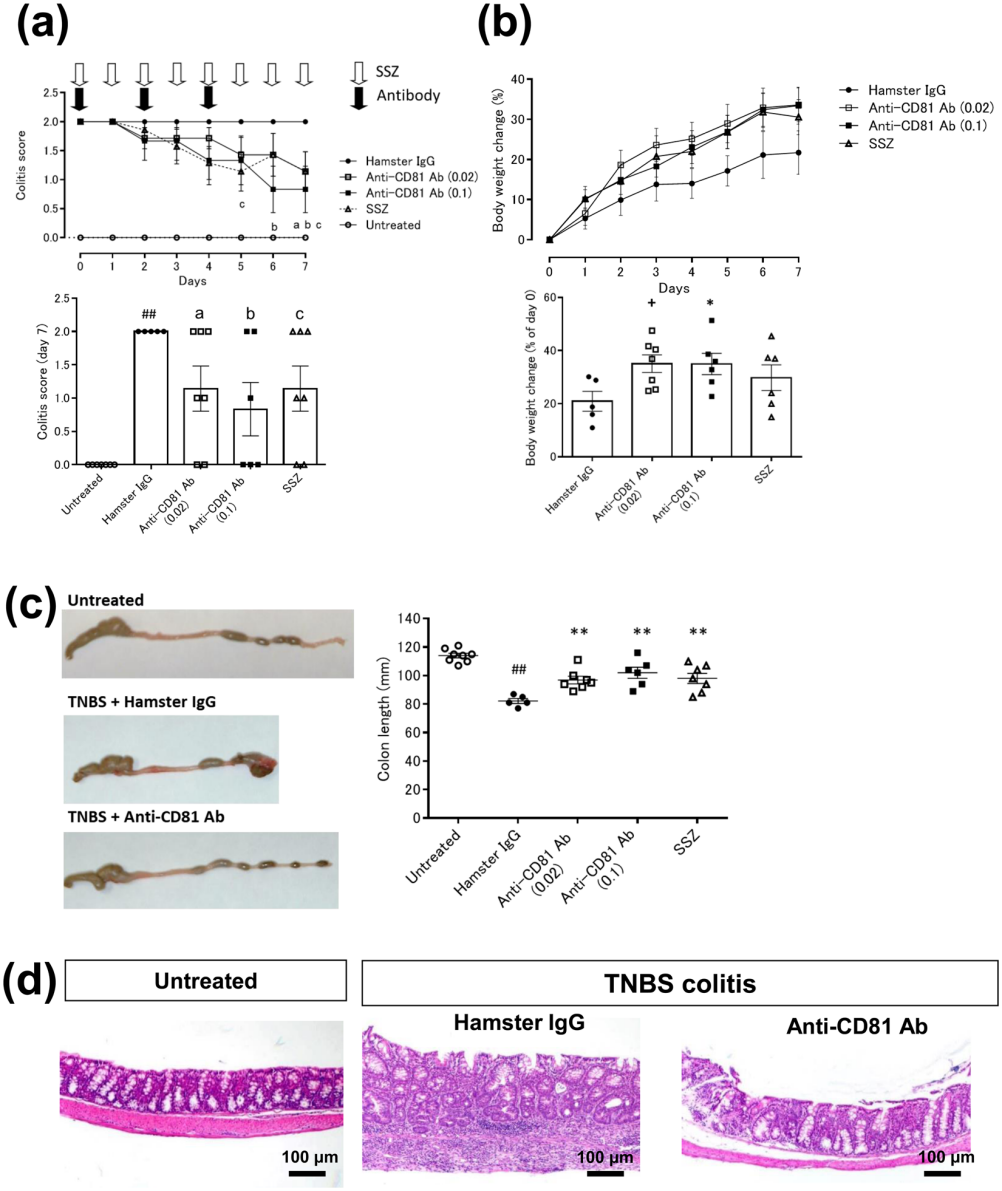
Short-term effects of the anti-CD81 antibody (2F7) on TNBS-induced colitis (7 days). Mice with established TNBS-induced colitis were divided into four groups on day 0. Colitic mice were injected intraperitoneally with hamster IgG and the anti-CD81 antibody. SSZ was administered orally. Open circle, untreated (n = 8); closed circle, TNBS-induced colitic mice treated with hamster IgG at 0.1 mg/mouse (n = 5) on days 0, 2, and 4; open and closed square, TNBS-induced colitic mice treated with the anti-CD81 antibody at 0.02 mg/mouse (n = 7) and 0.1 mg/mouse (n = 6) on days 0, 2, and 4; open triangle, TNBS-induced colitic mice treated with SSZ at 200 mg/kg (n = 7) orally administered from day 0 to 7. Data are representative of at least three independent experiments. (**a**) Colitis scores were evaluated daily (line graph). The bar graph is the average colitis score on day 7. Statistical significant was determined using Wilcoxon’s test [^##^*p* < 0.01, untreated vs hamster IgG; a: *p* < 0.05, hamster IgG vs anti-CD81 antibody (0.02); b: *p* < 0.05, hamster IgG vs anti-CD81 antibody (0.1); c: *p* < 0.05, hamster IgG vs SSZ]. (**b**) Body weights were measured daily (line graph). The bar graph shows the body weight change (% of day 0) on day 7. Statistical significance was determined using the Student’s t-test [*p < 0.05, hamster IgG vs anti-CD81 antibody (0.1); ^+^*p* < 0.05, hamster IgG vs anti-CD81 antibody (0.02)]. (**c**) Representative macroscopic images of the colon and colon length. Colons were removed on day 7 and their lengths measured. Statistical significance was determined using the Student’s t-test (^##^*p* < 0.01, untreated vs hamster IgG; **p < 0.01, hamster IgG vs anti-CD81 antibody, SSZ)). (**d**) Representative immunohistochemical staining of colons from treated and untreated mice with TNBS-induced colitis (×40). Hamster IgG or the anti-CD81 antibody was intraperitoneally injected into TNBS-induced colitic mice on day 0 and 2. Colons were removed on day 7, and sections were prepared for haematoxylin-eosin staining.

The effect of an anti-CD81 antibody on acute intestinal inflammation was examined in mice with acute colitis. Mice with established TNBS-induced colitis were administered the anti-mouse CD81 antibody (clone 2F7) and histopathological changes were examined for 7 days (Supplementary Fig. 1 and Table 1). Treatment with the anti-CD81 antibody on days 0, 2, and 4 and daily administration of sulfasalazine (SSZ) attenuated the colitis score. Notably, the colitis score on day 7 was significantly reduced by both the anti-CD81 antibody and SSZ compared with the vehicle group (Fig. 2A). Although there were eight mice with established colitis per group, one to three mice in each group died despite the reduction of the colitis score by anti-CD81 antibody and SSZ treatments. Mice with established TNBS-induced colitis treated with the anti-CD81 antibody had increased body weights compared with vehicle-treated mice, and the difference in the body weight change over 7 days was statistically significant (Fig. 2B). The length of the colons of mice with TNBS-induced colitis was shortened compared with that of untreated mice, and treatment with the anti-CD81 antibody and SSZ significantly improved the colon length (Fig. 2C). TNBS-induced colitis mice had inflamed and thickened colons, and anti-CD81 antibody treatment improved these pathological changes (Fig. 2D and Supplementary Fig. 2). Although the anti-CD81 antibody slightly decreased epithelial cell apoptosis (TdT-mediated dUTP Nick End Labelling+; TUNEL+) in the colon, it was not statistically significant (Supplementary Fig. 3). Inflammatory markers, including C-reactive protein (CRP), lymphotactin, monocyte chemoattractant protein-5, and macrophage inflammatory protein-2, were increased in the serum of mice with TNBS-induced colitis compared with untreated mice. The anti-CD81 antibody reduced the levels of these markers (Table 1). Thus, the anti-CD81 antibody had therapeutic effects on acute intestinal inflammation.

**Table 1:** Inflammation markers in serum from SJL/J mice with TNBS-induced colitis and untreated after 3 days of treatment with the anti-CD81 antibody (2F7, 0.5 mg/mouse) or control antibody.

	Untreated	TNBS-induced colitis	
		Hamster IgG	Anti-CD81 antibody
CRP	3.6 ± 0.2	6.3 ± 0.8**	4.1 ± 0.4^#^
Lymphotactin	126.8 ± 6.7	210.5 ± 19.2**	163.7 ± 6.4^#^
MCP-5	144.5 ± 0.9	185.8 ± 2.4**	118.8 ± 11.9^##^
MIP-2	13.4 ± 0.9	18.7 ± 1.6**	13.3 ± 1.5^#^

CRP, C-reactive protein; MCP-5, Monocyte chemoattractant protein-5; MIP-2, Macrophage inflammatory protein-2. Data are expressed as mean concentrations [pg/mL or μg/mL (CRP)] ± SE. n = 6. Data are representative of three independent experiments. Statistical significance was determined using the Student’s t-test. **p < 0.01 untreated vs. TNBS colitic mice treated with hamster IgG, ^#^*p* < 0.05, ^##^*p* < 0.01 hamster IgG vs. anti-CD81 antibody.

### Anti-CD81 antibody has long-lasting effects on TNBS-induced colitis in mice

**Figure 3:**
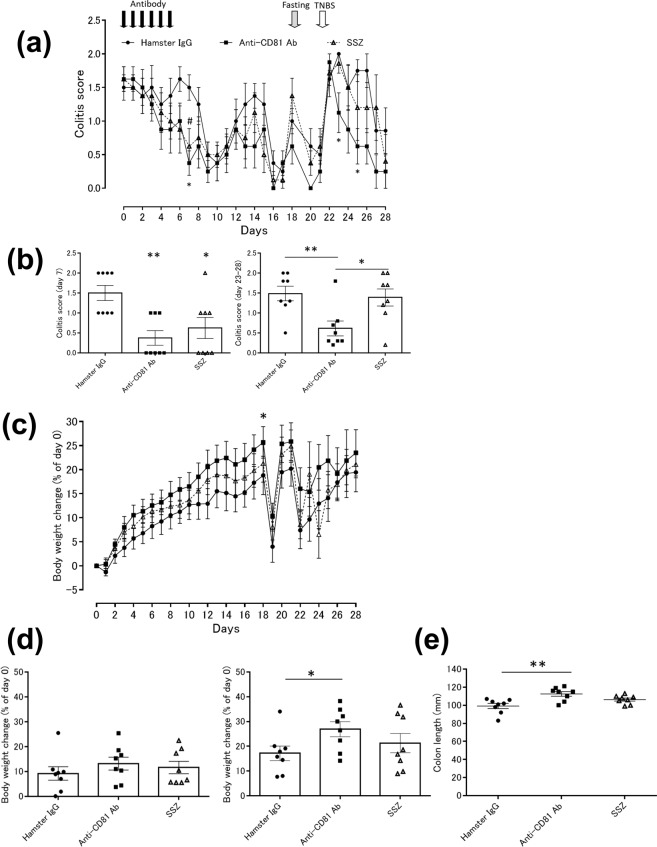
Anti-CD81 antibody (2F7) attenuates TNBS-induced colitis in the long term (28 days). Mice with established TNBS-induced colitis were divided into three groups on day 0. Colitic mice were injected intraperitoneally with hamster IgG or the anti-CD81 antibody. SSZ was administered orally. Mice were intrarectally infused with a TNBS solution on day 21. Closed circle, TNBS-induced colitic mice treated with hamster IgG at 0.1 mg/mouse for 6 days (day 0–5); closed square, TNBS-induced colitic mice treated with the anti-CD81 antibody at 0.1 mg/mouse for 6 days (day 0–5); open triangle, TNBS-induced colitic mice treated with SSZ at 200 mg/kg from day 0 to 21. (n = 8 per group). Data are representative of at least three independent experiments. (**a, b**) Colitis scores were evaluated daily (a, line graph). Colitis score on day 7 (b, left graph) and days 23–28 (b, right graph). Statistical significance was determined using Wilcoxon’s test (*p < 0.05, **p < 0.01). (**c, d**) Body weight was measured daily (c, line graph). Body weight changes (% of day 0) on day 7 (d, left graph) and day 18 (d, right graph) are shown. Statistical significance was determined using the Student’s t-test (*p < 0.05). (**e**) Colons were removed and their lengths measured on day 28. Statistical significance was determined using the Student’s t-test (**p < 0.01).

To further examine the effect of the anti-CD81 antibody on intestinal inflammation, mice with established TNBS-induced colitis were intraperitoneally injected with the anti-mouse CD81 antibody (clone 2F7) once a day for 6 days, and changes in the colitis score and body weight were examined for 28 days until colitis symptoms had diminished (Fig. 3, Supplementary Fig. 4a and Supplementary Table 1). Because colitis scores were reduced after 1 week, all mice were fasted on day 18 and then intrarectally re-administered the TNBS solution to induce a flare of colitis on day 21. Treatment with the anti-CD81 antibody significantly attenuated the colitis score on days 7, 23, and 25. Moreover, the anti-CD81 antibody attenuated the colitis score significantly from day 23 to 28 compared with SSZ. In contrast, SSZ reduced the colitis score on day 7, but not from day 23 to 28 (Fig. 3A, B). This experimental condition was milder than the previous experiment in Fig. 2, because 8-week body weights of mice with established colitis ranged from 16.9 to 23.0 g and all mice were still alive at day 21. The body weight change of mice with established TNBS-induced colitis treated with the anti-CD81 antibody did not reach statistical significance from day 0 to 7, but the body weight change from day 0 to 18 was increased significantly. In contrast, SSZ treatment did not influence the body weight change from day 0 to 7 or from day 0 to 18 (Fig. 3C, D). In addition, the anti-CD81 antibody improved the colon length compared with hamster IgG (Fig. 3E). Therefore, after stopping anti-CD81 antibody treatment, its therapeutic effect was maintained.

### Two different clones of the anti-mouse CD81 antibody are effective against TNBS-induced colitis

**Figure 4:**
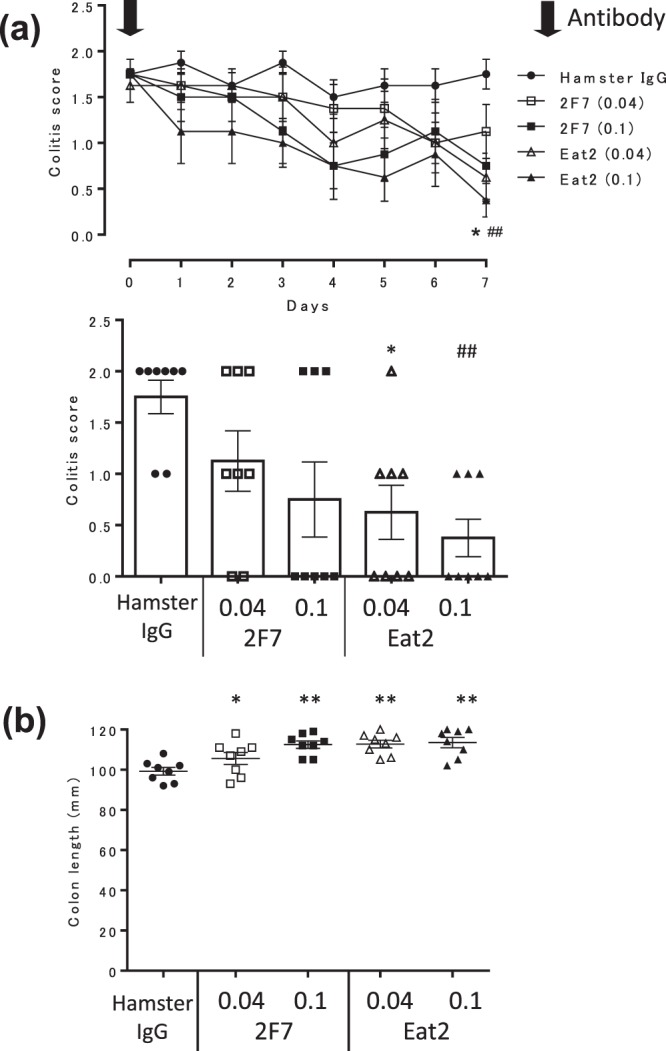
Therapeutic effects of clone Eat2 of the anti-mouse CD81 antibody on TNBS-induced colitis are superior to those of clone 2F7. Mice with established TNBS-induced colitis were divided into five groups on day 0 (n = 8 per group). Colitic mice were injected intraperitoneally with hamster IgG or the anti-CD81 antibody on day 0. Closed circle, TNBS-induced colitic mice treated with hamster IgG at 0.1 mg/mouse; open and closed square, TNBS-induced colitic mice treated with the anti-CD81 antibody (2F7) at 0.04 and 0.1 mg/mouse respectively; open and closed triangle, TNBS-induced colitic mice treated with the anti-CD81 antibody (Eat2) at 0.04 and 0.1 mg/mouse, respectively. Data are representative of at least three independent experiments. (**a**) Colitis scores were evaluated daily (line graph). Bar graph shows the average colitis score on day 7. Statistical significance was determined using Wilcoxon’s test (*p < 0.05, hamster IgG vs Eat2 (0.04); ^##^*p* < 0.01, hamster IgG vs Eat2 (0.1)). (**b**) Colons were removed and their lengths measured on day 7. Statistical significance was determined using the Student’s t-test (*p < 0.05, **p < 0.01, hamster IgG vs. anti-CD81 antibody).

To compare the effectiveness of different clones (2F7 and Eat2) of the anti-mouse CD81 antibody on intestinal inflammation, mice with TNBS-induced colitis were administered the clones only once on day 0. Clone Eat2 significantly attenuated colitis scores at doses of 0.1 and 0.04 mg/mouse. In contrast, the effect of clone 2F7 on colitis score was not significant when administered only once (Fig. 4A, Supplementary Fig. 4b, and Table 1). Both clones significantly improved the colon length. Mice treated with hamster IgG, 0.04 mg/mouse 2F7, 0.1 mg/mouse 2F7, 0.04 mg/mouse Eat2, or 0.1 mg/mouse Eat2 had colons with average lengths of 99.3 ± 1.9 mm, 112.5 ± 1.9 mm, 105.6 ± 3 mm, 113.0 ± 3.1 mm, and 112.8 ± 1.9 mm, respectively (Fig. 4B). In addition, another anti-CD81 antibody, Eat1, ameliorated TNBS-induced colitis (Supplementary Figs. 4c and 5 and Supplementary Table 1). Thus, multiple anti-mouse CD81 antibody clones had therapeutic effects on intestinal inflammation.

### Anti-CD81 antibody inhibits migration of T cells induced by human stromal cell-derived factor 1 (SDF-1)

**Figure 5:**
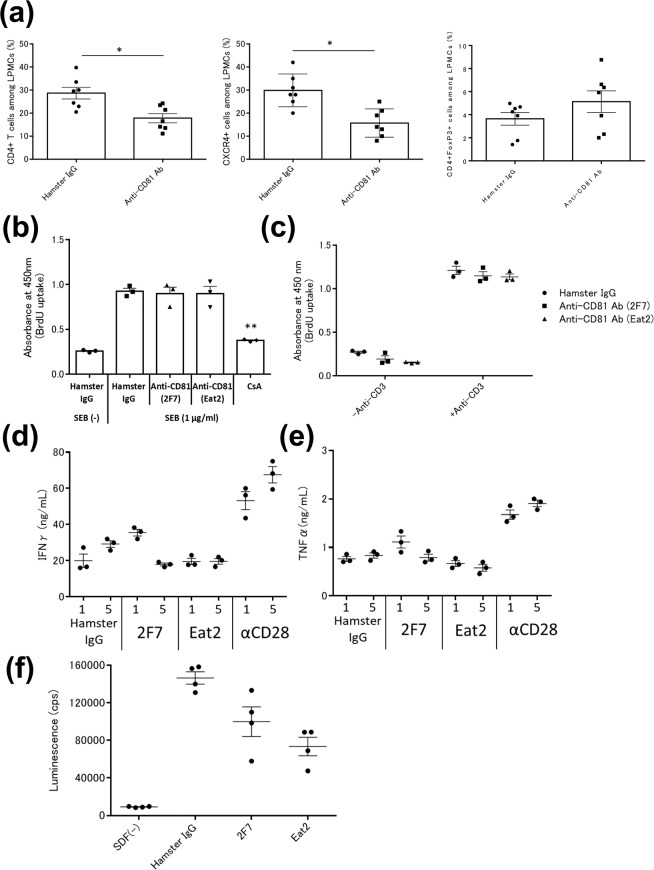
Anti-CD81 antibody reduces the frequency CD4+ T cells and inhibits migration induced by SDF-1. (**a**) Mice with established TNBS-colitis mice were divided into two groups and then intraperitoneally injected with hamster IgG or the anti-CD81 antibody Eat2 (0.1 mg/mouse, n = 7 per group). After 2 days, lamina propria mononuclear cells (LPMCs) were prepared and stained with anti-CD3ε, anti-CD4, anti-CXCR4, and anti-FoxP3 antibodies for flow cytometric analysis. Data are representative of three independent experiments. Statistical significance was determined using the Student’s t-test (*p < 0.05). (**b, c**) Splenocytes from mice with established TNBS colitis were cultured with hamster IgG, anti-CD81 (2F7 and Eat2) antibodies at 10 µg/mL, and CsA at 100 nM under stimulation by (**b**) 1 µg/mL SEB or (**c**) 5 µg/ml anti-CD3ε antibody. After 2 days, BrdU was added to the cultures and detected by an ELISA (n = 3 per group). Data are representative of at least three independent experiments. Statistical significance was determined using the Student’s t-test (**p < 0.01, hamster IgG vs anti-CD81 antibody, CsA) (**d, e**) Splenocytes from mice with established TNBS-induced colitis mice were prepared and cultured in 96-well plates precoated with 1 µg/ml anti-CD3ε antibody. Hamster IgG, anti-CD81 antibodies (2F7 and Eat2), and an anti-CD28 antibody at 5 µg/mL were added to the cultures, followed by 3 days of incubation. IFNγ (**d**) and TNFα (**e**) in culture supernatants were measured by ELISAs (n = 3 per group). Data are representative of at least three independent experiments. (**f**) Splenocytes from mice with established TNBS-induced colitis were prepared and cultured with hamster IgG or anti-CD81 antibodies (2F7 and Eat2) at 10 µg/mL for 2 h. Cells were seeded in the upper chamber of a 96-well plate containing 5-µm pore transwells with or without 10 ng/mL SDF-1 in the lower chamber for migration assays. The number of cells was counted by ATPlite (n = 4 per group). Data are representative of at least three independent experiments. Statistical significance was determined using the Student’s t-test (**p < 0.01, hamster IgG vs. anti-CD81 antibody).

Next, we investigated the mechanism of the effect of the anti-mouse CD81 antibody on TNBS-induced colitis, focusing on T cell functions using the clone Eat2. Colitic mice were administered Eat2 and changes in the frequency of T cells among colon lamina propria mononuclear cells (LPMCs) were determined at 2 days after administration. Although the frequencies of CD4^+^ T and CXCR4+ cells among LPMCs were decreased by Eat2, Eat2 did not affect the frequency of Treg (CD4+FoxP3+) cells (Fig. 5A). Furthermore, the therapeutic efficacy of Eat2 was associated with decreases in IL-1α and IL-6 levels detected in colonic homogenates from mice with TNBS colitis (Supplementary Fig. 6). Because CD81 was degraded by digestive enzymes in the preparation of colonic cells, the action of the anti-mouse CD81 antibody was determined on splenocytes and not colonic cells in mice with TNBS-induced colitis. However, CD69^+^ T cells from untreated mice with established TNBS-induced colitis were enriched for CD81^+^ T cells by 2.1–3.2-fold versus CD81^−^ T cells with or without staphylococcal enterotoxin B (SEB) stimulation (Supplementary Figs. 7–10). The anti-mouse CD81 antibody had no effect on the proliferation of splenocytes from mice with TNBS-induced colitis, which were stimulated with SEB or an immobilised anti-mouse CD3ε antibody (Fig. 5B, C). The anti-mouse CD81 antibody also had no influence on the cytokine production of splenocytes from mice with TNBS-induced colitis, which were stimulated with the immobilised anti-mouse CD3ε antibody or SEB (Fig. 5D, E). CXCR4^+^ T cells in mice with established TNBS-induced colitis were enriched among CD81^+^ T cells by 2.6- or 3.9-fold versus CD81^−^ T cells with or without SEB stimulation, respectively (Supplementary Figs. 7–10). The anti-CD81 antibody inhibited the migration of splenocytes from mice with TNBS-induced colitis, which was induced by SDF-1, one of the ligands for chemokine receptor CXCR4 and a chemokine involved in the pathogenesis of IBD (Fig. 5F)^1,8^. The anti-CD81 antibody also inhibited SDF-1-induced migration of mouse T cell line EL4.IL-2 (Supplementary Fig. 11)^9^. Thus, the anti-CD81 antibody reduced migration of activated T cells, which attenuated intestinal inflammation in the TNBS-induced model of colitis.

### Anti-CD81 antibody is also effective against sodium dextran sulphate salt (DSS)-induced colitis in mice

**Figure 6:**
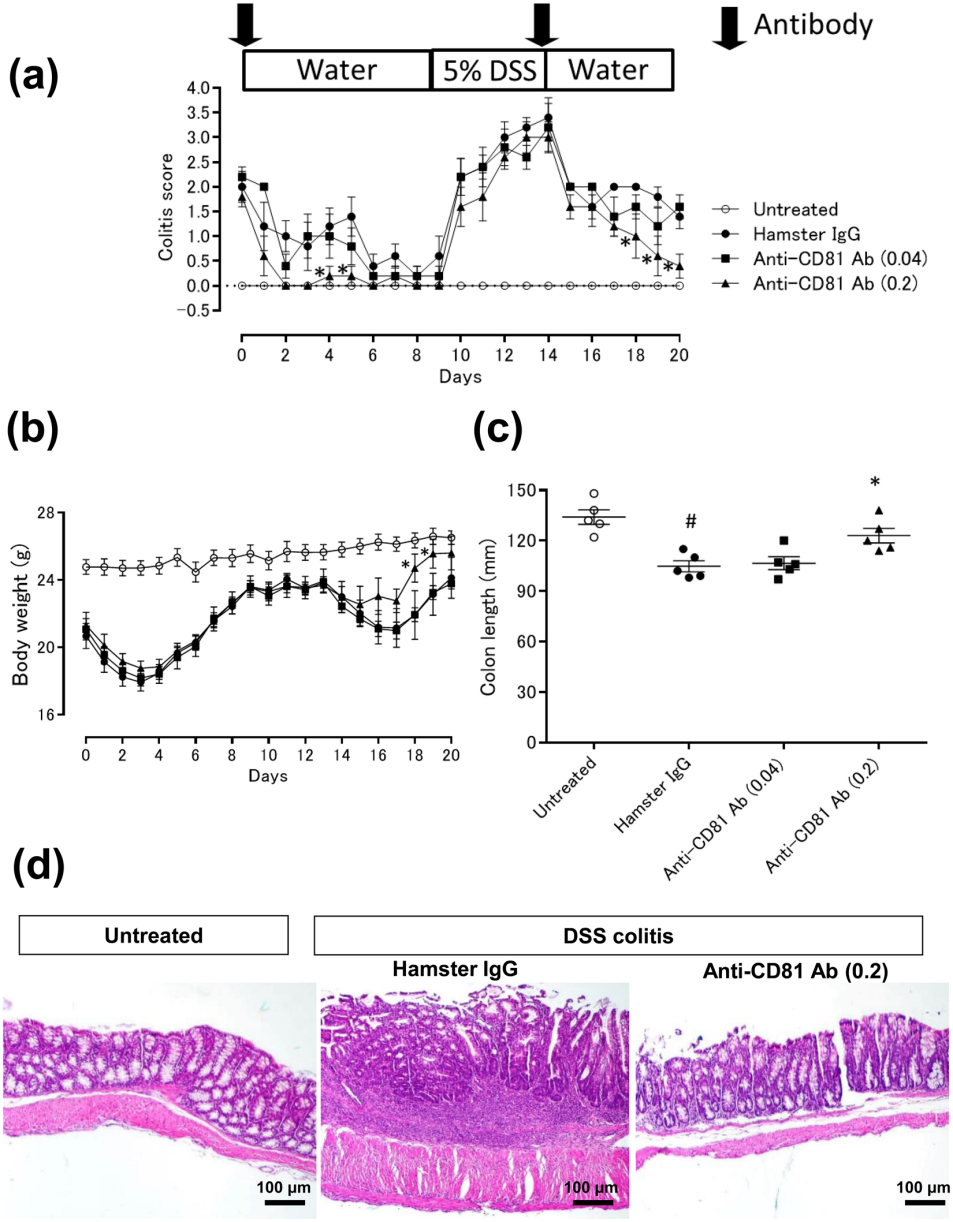
Anti-CD81 antibody (2F7) ameliorates DSS-induced colitis. Mice were administered 5% DSS in drinking water from day −5 to 0. Then, the mice were divided into three groups on day 0 (N = 5 per group). Colitic mice were injected intraperitoneally with hamster IgG or the anti-CD81 antibody on days 0 and 14. Open circle, untreated; closed circle, DSS-induced colitic mice treated with hamster IgG at 0.2 mg/mouse; closed square, DSS-induced colitic mice treated with the anti-CD81 antibody at 0.04 mg/mouse; closed triangle, DSS-induced colitic mice treated with the anti-CD81 antibody at 0.2 mg/mouse. Data are representative of at least three independent experiments. (**a**) Colitis scores were evaluated daily. Statistical significance was determined using Wilcoxon’s test (*p < 0.05, hamster IgG vs anti-CD81 antibody). (**b**) Body weights were measured daily. (**c**) Colons were removed and their lengths measured on day 20. Statistical significances of (**b, c**) were determined using the Student’s t-test (^#^*p* < 0.05, untreated vs hamster IgG; *p < 0.05, hamster IgG vs anti-CD81 antibody). (**d**) Representative immunohistochemical staining of colons from treated and untreated mice with DSS-induced colitis (×40). Hamster IgG or the anti-CD81 antibody was intraperitoneally injected into DSS-induced colitic mice on days 0 and 14. Colons were removed on day 20, and sections were prepared for haematoxylin-eosin staining.

Finally, we assessed the therapeutic potential of the anti-CD81 antibody in another colitis model, DSS-induced colitis. Mice with DSS-induced colitis were administered the anti-mouse CD81 antibody (clone 2F7) on days 0 and 14. Treatment with the anti-CD81 antibody at 0.2 mg/mouse significantly reduced the colitis score not only on days 4 and 5, but also from day 18 to 20 compared with the vehicle group (Fig. 6A). In addition, mice with DSS-induced colitis and treated with the anti-CD81 antibody at 0.2 mg/mouse had improved body weights on days 18 and 19 compared with vehicle-treated mice (Fig. 6B). The length of the colons in mice with DSS-induced colitis was shortened compared with those in untreated mice, and treatment with the anti-CD81 antibody at 0.2 mg/mouse significantly improved the colon length (Fig. 6C). Histological analysis of DSS-induced colitis mice showed features of colitis, such as depletion of mucus and cell infiltration in the mucosa, and the anti-CD81 antibody treatment improved these pathological changes (Fig. 6D). Thus, the anti-CD81 antibody had therapeutic effects on DSS-induced acute intestinal inflammation.

## Discussion

In this study, we examined the contribution of CD81 to IBD pathophysiology. CD81 has been reported to be expressed on multiple kinds of cells at various expression level and have roles in proliferation, differentiation, cytokine production, migration, and other biological actions^10^. We found increased CD81 expression on T cells in peripheral blood and Peyer’s patches of mice with TNBS-induced colitis. An endogenous ligand for CD81 has not been identified, but its signaling is involved in forming complexes consisting of polarised patches of increased CD81 molecules and other associated molecules^3^. For example, Mittelbrunn *et al*. reported that immune synapse formation induces CD81 polarisation on T cells and APCs^6^. A prominent increase in CD81 expression on activated T cells suggests that CD81 contributes to the functions of T cells. We showed that the anti-mouse CD81 antibody improved colitic symptoms, including shortened inflamed colons and reduced body weight, and attenuated histological changes in colons and inflammatory markers in the blood of mice with acute TNBS-induced colitis. Moreover, the anti-CD81 antibody improved colitic symptoms and body weight change of mice with a flare-up of TNBS-induced colitis after stopping anti-CD81 antibody treatment. Interestingly, it was previously shown that CD81 induces quiescence of hematopoietic stem cells^11^. This function of CD81 may be involved in the reset of inflammatory lymphocytes in colitis and contribute to the long-lasting effect of anti-CD81 antibodies. Because IBD is a chronic remitting/relapsing disease, the long-term effects of the anti-CD81 antibody would be particularly useful for IBD therapy.

Dijkstra and colleagues demonstrated that the anti-mouse CD81 antibody clone 2F7 is not effective against experimental autoimmune encephalomyelitis, although another clone, Eat2, is therapeutic^12^. Different clones of an anti-CD81 antibody exert different effects on biological functions^10^. We showed that two different anti-mouse CD81 antibody clones, 2F7 and Eat2, were both effective against colitis, although Eat2 was more effective than 2F7. In addition, as another clone of anti-CD81 antibody, Eat1 was also effective against TNBS-induced colitis. Therefore, we hypothesised that CD81 regulated TNBS-induced colitis. We examined the mechanism underlying the therapeutic effect of the anti-CD81 antibody and found that CD4^+^ T cells were decreased in colons of mice with colitis treated with the anti-CD81 antibody. Although anti-CD81 antibodies had no influence on the proliferation or cytokine release of stimulated lymphocytes from mice with colitis, the anti-CD81 antibody reduced migration of lymphocytes from both colitic mice and that of a mouse T cell line towards SDF-1. IFNγ^+^ T cells in established TNBS-colitis mice were rich in CD81^+^ T cells at 3.7-fold (12.8% versus 3.5%) or 4.9-fold (6.4% versus 1.3%) compared with CD81^−^ T cells with or without SEB stimulation, respectively (Supplementary Figs. 7 and 9). IL-17^+^ T cells from mice with established TNBS-induced colitis were rich in CD81^+^ T cells at 3.7-fold (11.9% versus 3.2%) and 19-fold (3.8% versus 0.2%) compared with CD81^−^ T cells with or without SEB stimulation, respectively (Supplementary Figs. 7 and 9). These results indicated that CD81^+^ T cells were mostly effector T cells in TNBS-induced colitis^13^. Therefore, it was likely that the anti-CD81 antibody reduced migration of effector T cells in mice with colitis and improved colonic inflammation. However, because this inhibitory effect was not complete, other mechanisms may contribute to the therapeutic effect of the anti-CD81 antibody on murine colitis. Recently, it was reported that CD81 knockout mice have impaired functions of regulatory T cells in a tumour transplantation model^14^.

Finally, we confirmed the therapeutic potential of the anti-CD81 antibody in another model, DSS induced colitis. The anti-CD81 antibody ameliorated the colitis score and improved colon lengths in mice with DSS-induced colitis. Although there are potential limitations in IBD animal models, such as species differences and incomplete disease manifestations, we have shown that CD81 plays a critical role in IBD pathophysiology. Because CD81 is a critical or modified molecule for various biological functions, such as cell migration, resetting the cell cycle, and Treg functions, complete understanding of the mechanism of the therapeutic effect of anti-CD81 antibodies on IBD is necessary prior to implementation of CD81-targeted therapy in the clinic.

## Materials and methods

### Mice

Four to 6-week-old male SJL/J mice (Charles River Japan, Japan) and 6-week-old male BALB/cAnNCrlCrlj (Charles River Japan, Japan) were purchased and housed under specific pathogen-free conditions. Mice were quarantined and acclimatised for 1 week before use and then housed in a controlled environment (23 ± 2 °C; 55 ± 10% humidity) with a 12-hour light/dark cycle (lights on at 8:00 AM) and allowed free access to food (CE-2; Clea Japan, Inc., Japan) and filtered water. All study protocols were approved by the Animal Care and Use Committee of Sumitomo Dainippon Pharma Co., Ltd., and undertaken in accordance with the regulations for animal experiments in this division.

### Antibodies

A hamster anti-mouse CD81 antibody (2F7) was purchased from Southern Biotech (UK). Anti-mouse CD81 antibody (Eat1) was purchased from Santa Cruz (US). Hamster IgG (HTK888), anti-mouse CD81 (Eat2), biotin-anti-mouse CD81 (Eat2), FITC-anti-hamster IgG (poly4055), FITC-anti-mouse CD69 (H1.2F3), isotype-matched FITC-hamster IgG (HTK888), PE-anti-mouse CD81 (Eat2), isotype-matched PE-hamster IgG (HTK888), and PerCP-streptavidin were obtained from Biolegend (USA). APC-anti-mouse CD4 antibodies (GK1.5), isotype matched APC-rat IgG2b (RTK2071), PE-anti-mouse FoxP3(FJK-16s), an APC-anti-mouse CD3ε (145–2C11) antibody, and APC-hamster IgG (eBio299Arm) were purchased from eBioscience (USA). FITC-anti-mouse CD3ε (145–2C11), anti-mouse CD3ε (145–2C11), anti-mouse CD28 (37.51), PE-anti-mouse CD69 (H1.2F3), PE-anti-mouse CXCR4 (2B11), PE-anti-mouse IFNγ (XMG1.2), PE-anti-mouse IL-17 (TC11–18H10), and anti-mouse CD16/32 antibodies (Fc block, 24 G.2) were purchased from BD Bioscience (USA).

### T cell culture and activation

PBMCs were prepared using Lympholyte-Mammals (CEDARLANE, Canada). PBMCs (2.5 × 10^6^/well in 6-well plates) were cultured with PHA (5 µg/ml; WAKO) and IL-2 (50 ng/ml; R&D Systems) in RPMI-1640 medium (Gibco) containing 10% fetal bovine serum (FBS) and penicillin-streptomycin at 37 °C in a humidified atmosphere containing 5% CO_2_ for 0, 24, 48, and 72 h.

### Flow cytometry analysis

PBMCs were prepared using Lympholyte-Mammals (CEDARLANE, Canada). Colon lamina propria mononuclear cells (LPMCs) were prepared according to previously described methods^15,16^. Briefly, colons were cut into pieces, digested with 1 mg/mL collagenase A (Roche, Switzerland) and 0.01% DNase (Sigma-Aldrich) in 1.5% FBS in Hank’s balanced salt solution (Invitrogen, USA) and then purified from the interface between a 75% and 40% Percoll gradient. Peyer’s patches, mesenteric lymph nodes, and spleens were ground with white edge glasses (Matsunami Glass, Japan). Splenocytes were treated with ACL lysis buffer (Gibco, USA) to remove red blood cells. The cells were filtered through a 70-µm pore size mesh to remove tissue debris. PBMCs were cultured in the absence or presence of 5 µg/ml PHA and 50 ng/ml recombinant human IL-2 (R&D Systems) in RPMI-1640 medium (Gibco) containing 10% FBS and penicillin-streptomycin. For intracellular staining, cells were cultured with or without 1 µg/mL SEB (sigma) for 50 h. The cells were treated with Golgi Stop (BD Bioscience) for 14 h. The cells were then incubated with an anti-mouse CD16/32 antibody for 20 min to block IgG Fc receptor binding and then stained with fluorescently labelled antibodies against cell surface markers (CD3, CD69, CD81, CXCR4, IFNγ, and IL-17). Stained cells were fixed with 4% paraformaldehyde and analysed on a FACSCalibur or FACSCanto II (BD Bioscience). Dead cells were excluded by forward light scatter and 7-aminoactinomycin D staining. In the lymphoid population, 2 × 10^4^ cells/sample were analysed. The threshold of positivity was defined as beyond the nonspecific binding observed in the presence of a relevant isotype control antibody.

### Induction of TNBS colitis

SJL/J mice were subcutaneously injected with an emulsion of 2,4,6-trinitrobenzene sulfonic acid (TNBS; Nacalai Tesque Inc., Japan) bound to ovalbumin (OVA; Sigma, USA) in complete Freund’s adjuvant (CFA; Difco Laboratories, USA). One week later, the mice were anesthetised and infused with 2 mg TNBS in 0.2 ml of 50% ethanol at 3 cm from the anus. To ensure TNBS distribution within the entire colon, the mice were held in a vertical position for 3 minutes after the infusion. Colitis scores were estimated by stool conditions: score 0, normal stool that was dry or wet, but maintained shape after prodding; score 1, loose stool that was wet and soft, and its shape was easily broken by prodding; score 2, diarrhoea. From 5 to 7 days after rectal infusion of TNBS, mice with colitis score 1 or 2 were determined as having established colitis. Colitic mice were divided into three or four groups by their body weight and stool score on day 0 using the “Block randomization with multiple variables” program in Stat Preclinica. Mice were sacrificed on day 7 for the short-term analysis or day 28 for the long-term analysis after the first anti-CD81 antibody administration, and then spleens, colons, and blood were collected for analyses. Serum from mice with colitis was analysed for C-reactive protein, lymphotactin, monocyte chemotactic protein-5 (MCP-5), and macrophage inflammatory protein-2 (MIP-2) using rodents multi-analyte profiles (Charles River Laboratories, Japan). Colons were removed, and sections were prepared for haematoxylin-eosin staining. Analysis of the levels of cytokines IL-1α and IL-6 in colon homogenates was performed by ELISAs (Invitrogen, USA).

### Immunohistochemistry

Colons were removed from mice, opened longitudinally, and fixed in 10% formaldehyde neutral buffer solution (Nacalai Tesque Inc.). Samples were embedded in paraffin and cut into 5 µm-thick sections. The sections were de-paraffinized, blocked, and incubated with a horseradish peroxidase-conjugated anti-mouse CD81 antibody (clone Eat2). Antibody binding was detected with DAB substrate using the Rabbit ImmunoCruz Staining System (Santa Cruz Biotechnology, USA) and counterstained with haematoxylin (Wako). All sections were observed by light microscopy.

### Anti-mouse CD81 Abs, hamster IgG, and SSZ treatment of TNBS-induced colitic mice for short-and long-term analyses

To examine the effect of anti-CD81 antibodies on colitis, mice with established colitis were randomised by colitis scores and body weights, grouped, and intraperitoneally injected with an anti-CD81 antibody (clone 2F7 or Eat2) or control hamster IgG. In the short-term effect analysis, mice received the antibody once a day on days 0, 2, and 4 (Fig. 2A–C), days 0 and 2 (Fig. 2D and Supplementary Fig. 2), or day 0 (Table 1, Figs. 4 and 5, Supplementary Figs. 3, 5, 6, and 12). In the long-term effect analysis, mice received the antibody once a day from day 0 to 5 (Fig. 3). SSZ (Sigma-Aldrich) was administered each day orally for the long-term analysis. Then, colons were collected for analyses.

### Proliferation and cytokine production of lymphoid cells from TNBS-induced colitic mice

Spleens were removed from mice with established TNBS-induced colitis and ground with white edge glasses. Splenocytes were treated with ACK lysing buffer to lyse red blood cells. Remaining cells were filtered through a 70-µm pore size mesh to remove debris. The prepared cells were seeded at 1 × 10^6^ cells/well in 96-well plates precoated with 5 µg/ml anti-mouse CD3ε antibody or 1 µg/ml SEB. After culture with hamster IgG, anti-CD81 antibodies (2F7 and Eat2), and an anti-CD28 antibody at 5 µg/mL each, and cyclosporine A (CsA, Sigma) at 100 nM for 3 days, cell proliferation was measured using the Biotrack ELISA system, version 2, containing BrdU and an anti-BrdU antibody (Amersham, UK) at an absorbance of 450 nm. Culture supernatants were also collected, and IFNγ and TNFα were measured by ELISAs (R&D Systems). Spleen cells from mice with TNBS-induced colitis were stimulated with or without 1 µg/mL SEB for 50 h. Cultured cells were treated with Golgi Stop for 14 h, and then cell surface molecules (CD69 and CXCR4) and intracellular cytokines (IFNγ and IL-17) were stained and analysed by the FACSCanto II.

### Migration assay

Splenocytes from mice with established TNBS-induced colitis or mouse T cell line EL4.IL-2 (ATCC, TIB-181) were prepared and incubated with hamster IgG and the anti-mouse CD81 antibody. A 75 µl cell suspension of 2 × 10^7^ cells/ml was added to the upper well of an HTS Transwell 96 well permeable support, 5 µm pore (Corning, USA) with or without 235 µl of 10 ng/ml recombinant human SDF-1 (PEPROTECH, UK) in the lower well. After 2 h of incubation at 37 °C with 5% CO_2_, 50 µl/well of the cell suspension in the lower well was transferred to an EIA/RIA 96 well plate (Corning) and 50 µl/well ATPlite (PerkinElmer, USA) was added to each well. Luminescence was measured using an Envision 2102 multilabel reader (PerkinElmer, USA).

### Induction of DSS colitis

BALB/cAnNCrlCrlj mice were administered 5% sodium dextran sulfate salt (molecular weight: 1,000–9,000; Wako Pure Chemicals, Japan) in drinking water from day −5 to 0. Colitis scores were estimated by stool conditions: score 0, normal stool that was dry or wet, but maintained shape after prodding; score 1, loose stool without bloody stool or a bloody bowel discharge or bloody stool without loose stool or diarrhoea; score 2, diarrhoea without bloody stool or a bloody bowel discharge or bloody bowel discharge without loose stool or diarrhoea; score 3, loose stool and a bloody bowel discharge or diarrhoea and bloody stool; score 4, diarrhoea and a bloody bowel discharge. On day 0, mice with a body weight decrease at day −1 of more than 20% compared with day −5 and mice with a colitis score of “0” were excluded from the study. The remaining mice were divided to three groups by their body weight and colitis score on day 0 using the “Block randomization with multiple variables” program in Stat Preclinica. Then, mice were administered distilled water from day 0 to 9, followed by 5 days of 5% DSS in their drinking water and 5 days of distilled water administration. Control animals were administered distilled water only. The mice were monitored daily for survival, body weight, and stool consistency. Then, colons were collected on day 20 for analyses.

### Statistical analysis

Data are expressed as means ± standard error (X ± SE). The statistical difference of colitis scores between groups was calculated by Wilcoxon’s test. The unpaired Student’s *t*-test was used to calculate the statistical significance of differences in colon length, body weight change, serum markers, migration assay, and frequency of colonic cell populations between two groups. Statistical analysis was performed using Stat Preclinica version 1.0.3295 (Takumi Information Technology Inc., Japan).

## Supplementary information

Supplymentary information

## Data Availability

All data generated or analysed during this study are included in this published article (and its Supplementary Information files).
