# Supplementary material for: Anti-CD81 antibodies reduce migration of activated T lymphocytes and attenuate mouse experimental colitis
Source: Sci Rep. 2020 Apr 24;10:6969. doi: 10.1038/s41598-020-64012-5 (PMC7181603; doi:10.1038/s41598-020-64012-5)
Supplement: Supplementary file 1 — Supplymentary information [file 41598_2020_64012_MOESM1_ESM.pdf]

Anti-CD81 antibodies reduce migration of activated T lymphocytes and attenuate mouse experimental colitis

Takuya Hasezaki[1,*], Tadahiko Yoshima[2] and Yukiko Mine[3]
1 External Innovation, Sumitomo Dainippon Pharma Co., Ltd, Osaka 554-0022, Japan
2 Applied Bioscience Group, Bioscience Research Laboratory, Sumitomo Chemical Co., Ltd, Osaka 554-0022, Japan
3 Group 2, Platform Technology Research Unit, Sumitomo Dainippon Pharma Co., Ltd, Osaka 554-0022, Japan

*Correspondence and request for materials should be addressed to Takuya Hasezaki
3-1-98 Kasugade Naka, Konohana-ku, Osaka 554-0022, Japan
Tel: +81-6-6466-5942
Fax: +81-6-6466-3457
Email: takuya-hasezaki@ds-pharma.co.jp

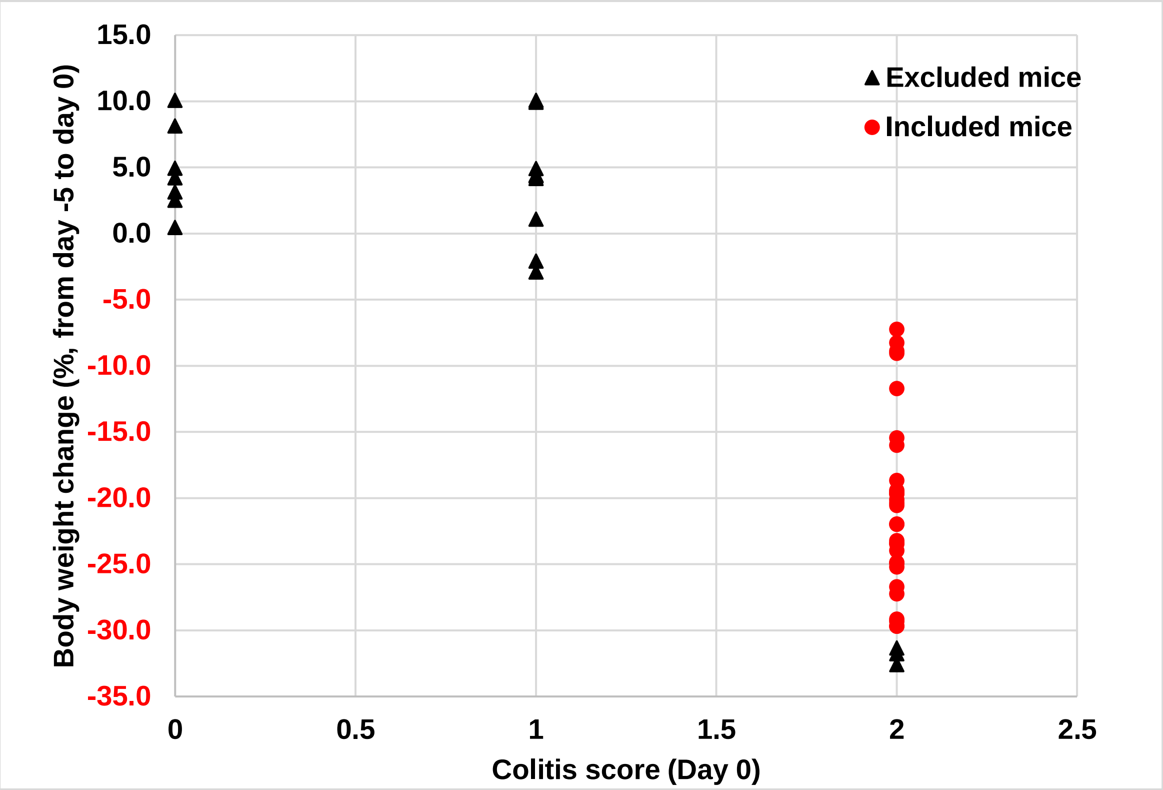

Supplementary Figure 1. Body weight change from TNBS induction (day -5) to grouping (day 0) and the colitis score on day 0 of Figure 2. TNBS was administered intrarectally on day -5. Body weight was measured on days -5 and 0. The body weight change (%) and colitis score on day 0 were plotted. Closed triangle, mice excluded from the study; closed circle, mice included in the study. Data are representative of at least three independent experiments.

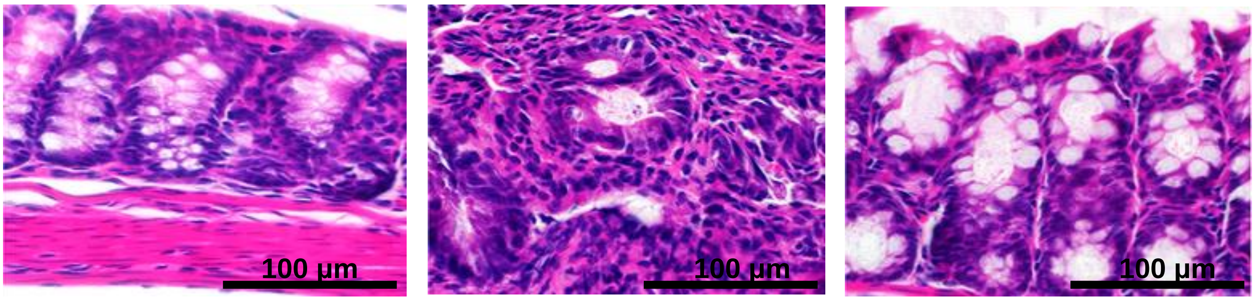

Supplementary Figure 2. Representative immunohistochemical staining of colons from anti-CD81 antibody (2F7) treated and untreated mice with TNBS-induced colitis. Mice with established TNBS-induced colitis were divided into four groups on day 0. Hamster IgG or the anti-CD81 antibody was intraperitoneally injected into TNBS-induced colitic mice on day 0 and 2. Colons were removed on day 7, and sections were prepared for haematoxylin-eosin staining (×400).

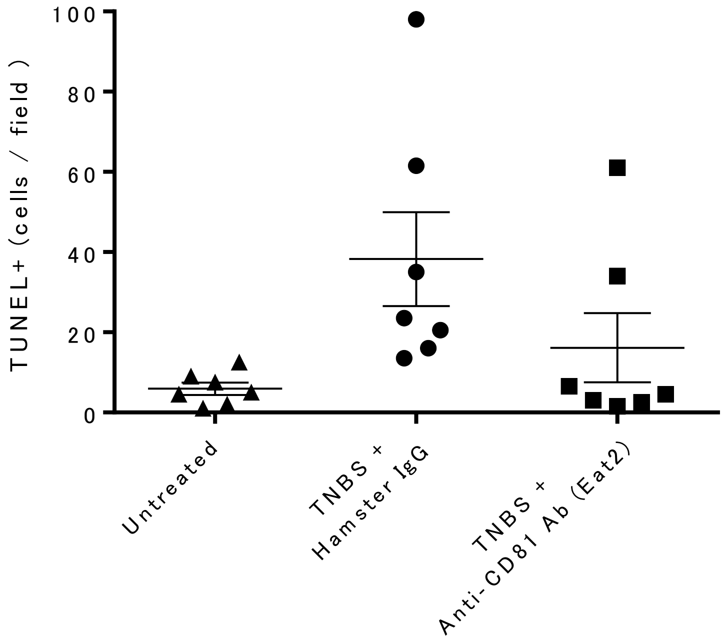

Supplementary Figure 3. Effect of the anti-CD81 antibody on TNBS-induced epithelial cell apoptosis of colons. TNBS was administered intrarectally on day -5. Colitic mice were injected intraperitoneally with hamster IgG at 0.5 mg/mouse and the anti-CD81 antibody at 0.5 mg/mouse on day 0 (n=7, per group). Colons were removed on day 7, and TUNEL staining was performed with an Apop Tag® Fluorescein in situ Apoptosis Detection kit, according to the manufacturer's instructions. Data are representative of two independent experiments.

a

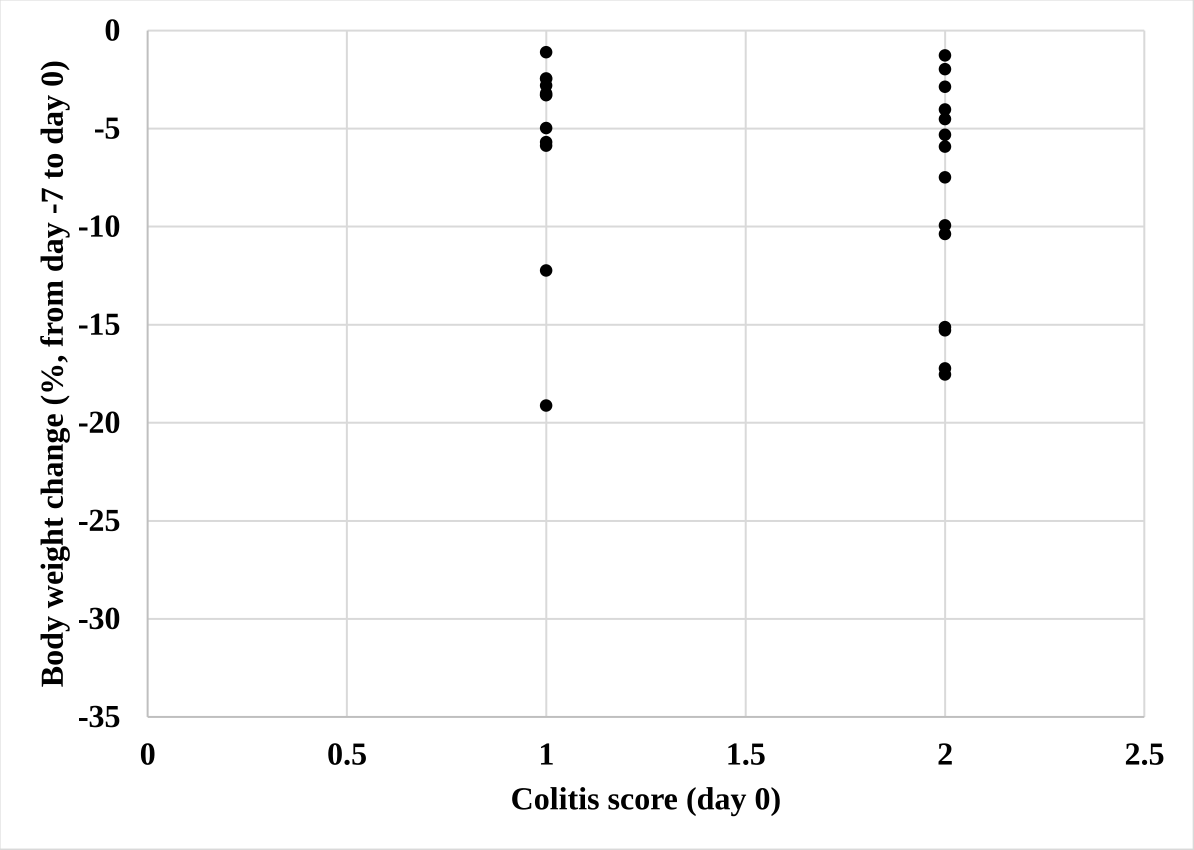
Body weight change (%, from day -7 to day 0)
0
-5
-10
-15
-20
-25
-30
-35
0
0.5
1
1.5
2
2.5
Colitis score (day 0)

b

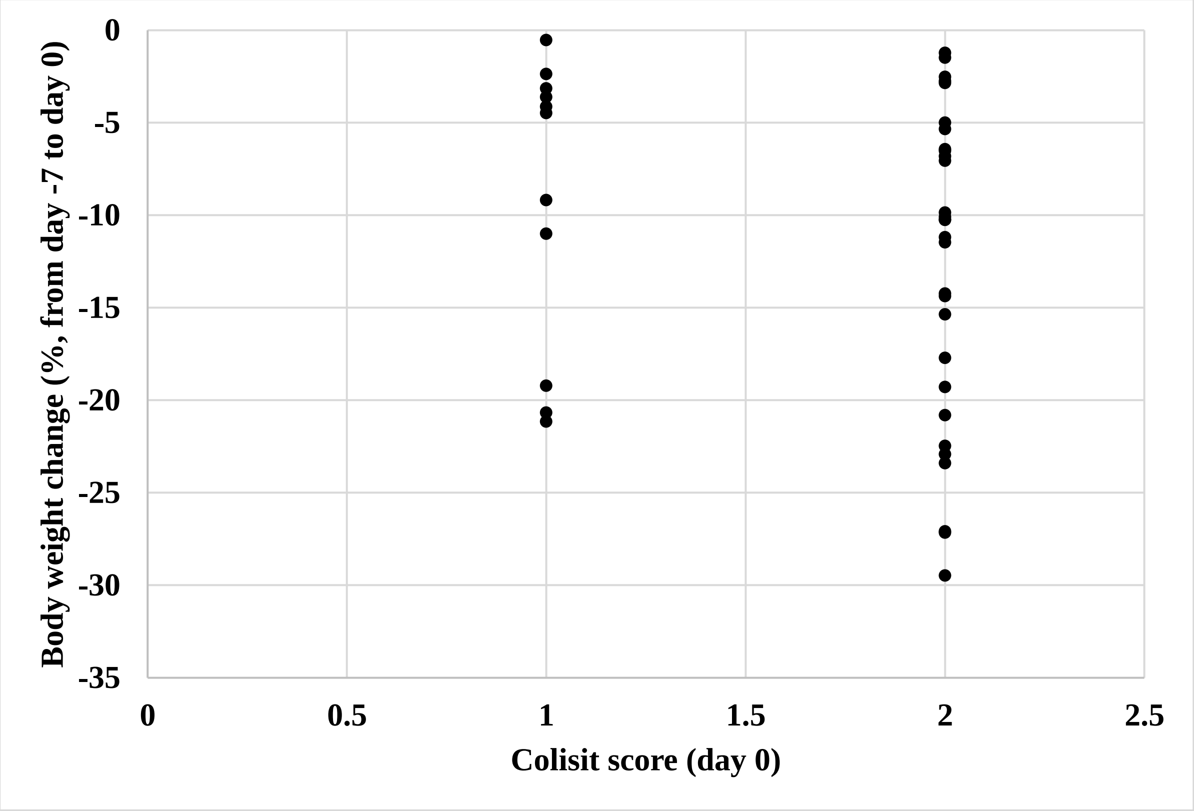
Body weight change (%, from day -7 to day 0)
0
-5
-10
-15
-20
-25
-30
-35
0
0.5
1
1.5
2
2.5
Colisit score (day 0)

c

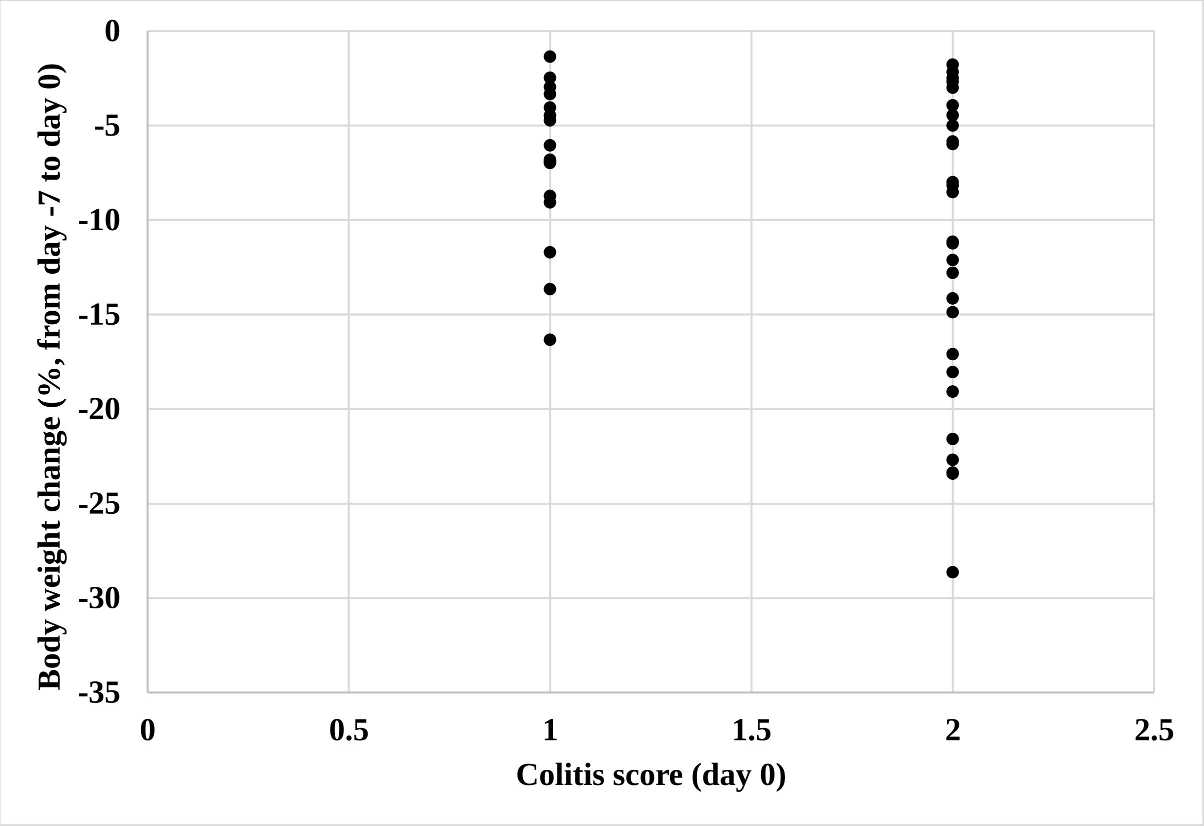

Supplementary Figure 4. Body weight change from TNBS induction to grouping (day 0) and the colitis score on day 0 of Figure 3, Figure 4, and Supplementary Figure 5. TNBS was administered intrarectally on day -7. Body weight was measured on days -7 and 0. The body weight change (%) and colitis score on day 0 of a, Figure 2, b, Figure 3, and c, Supplementary Figure 5 were plotted.

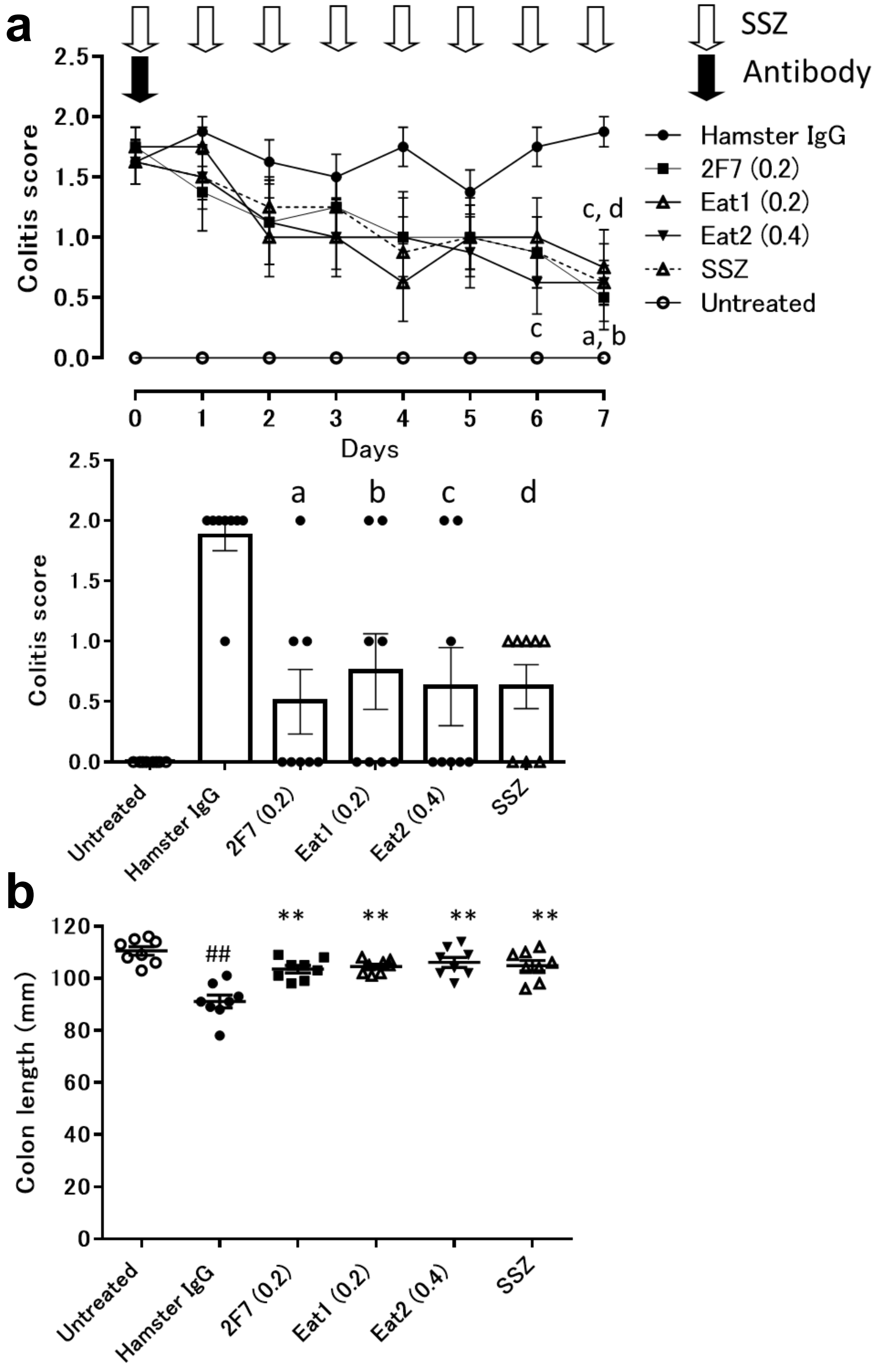

a
SSZ
Antibody
Colitis score
Days
Hamster IgG
2F7 (0.2)
Eat1 (0.2)
Eat2 (0.4)
SSZ
Untreated
c, d
c
a, b
a
b
c
d
Untreated
Hamster IgG
2F7 (0.2)
Eat1 (0.2)
Eat2 (0.4)
SSZ
b
Colon length (mm)
##
**

Supplementary Figure 5. Clones 2F7, Eat1, and Eat2 of the anti-mouse CD81 antibody are effective against TNBS-induced colitis. Mice with established TNBS-induced colitis were divided into five groups on day 0. Hamster IgG (0.4 mg/mouse) and three clones (2F7, Eat1, and Eat2) of the anti-CD81 antibody (0.2–0.4 mg/mouse) were injected intraperitoneally once on day 0. Sulfasalazine (SSZ) at 200 mg/kg was administered orally from day 0 to 7 (N=8 per group). Data are representative of three independent experiments. (a) Colitis scores were evaluated daily (line graph). The bar graph is the average colitis score on day 7. Statistical significance was determined using Wilcoxon's test (a: $p<0.05$, hamster IgG vs 2F7; b: $p < 0.05$, hamster IgG vs Eat1; c: $p < 0.05$, hamster IgG vs Eat2; d: $p < 0.05$, hamster IgG vs SSZ). (b) Colons were removed on day 7 and their lengths measured. Statistical significance was determined using the Student's t-test ($^{\#\#}p < 0.01$, untreated vs hamster IgG; $^{**}p < 0.01$, hamster IgG vs anti-CD81 antibody, SSZ).

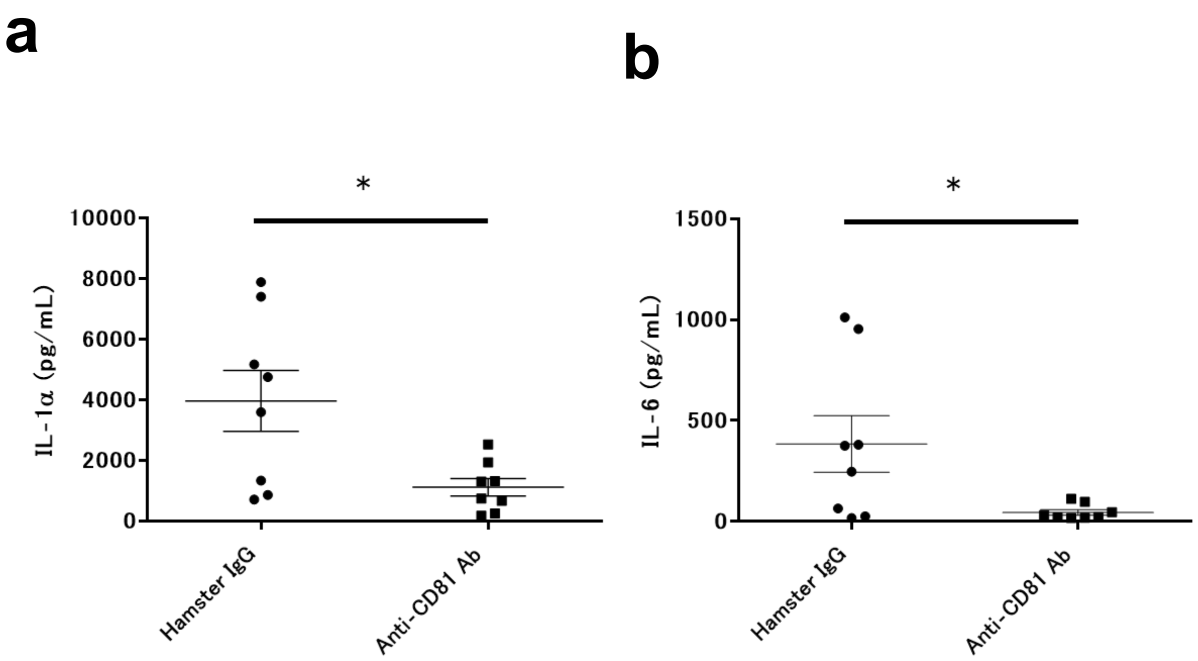

Supplementary Figure 6. Anti-CD81 antibody (Eat2) decreases inflammatory cytokines in the colon of mice with TNBS-induced colitis. TNBS was administered intrarectally on day -5. Colitic mice were injected intraperitoneally with hamster IgG at 0.5 mg/mouse and the anti-CD81 antibody (Eat2) at 0.5 mg/mouse on day 0 (n=8, per group). Colons were removed on day 4. Colonic homogenate levels of IL-1α (a) and IL-6 (b) were measured by ELISAs. Data are representative of two independent experiments. Statistical significance was determined using the Student's t-test (*$p < 0.05$, hamster IgG vs anti-CD81 antibody).

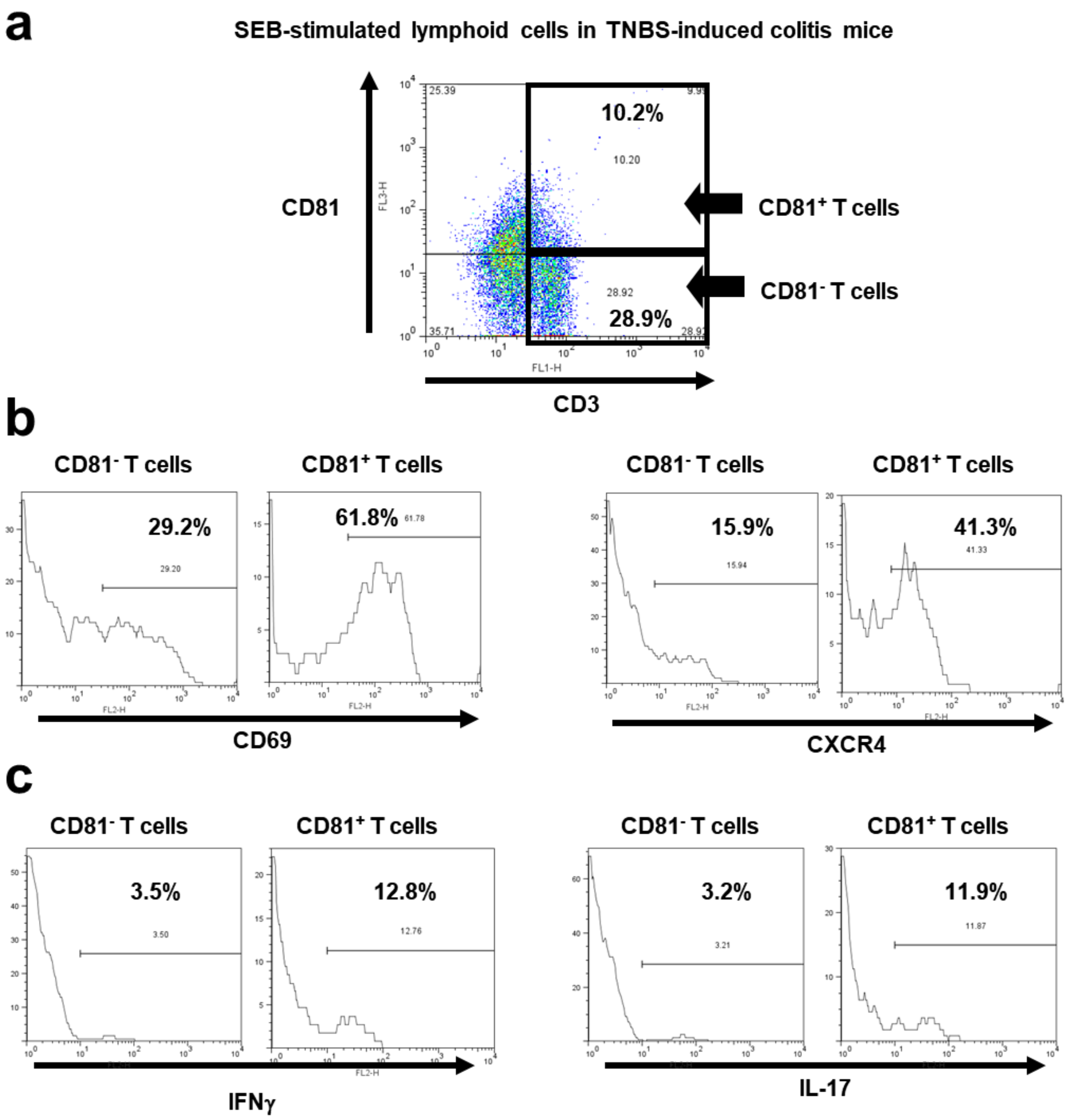

Supplementary Figure 7. CD81^{+} T cells in spleens of TNBS-induced colitis mice are active, migrate, and cytokine-producing effector T cells. Splenocytes from mice with TNBS-induced colitis mice were stimulated with 1 μg/mL staphylococcal enterotoxin B (SEB) for 50 h. The cells were treated with Golgi Stop for 14 h. (a, b) Cell surface molecules (CD81, CD69, and CXCR4) and (c) intracellular cytokines (IFNγ and IL-17) were then stained and analysed by a FACSCanto II.

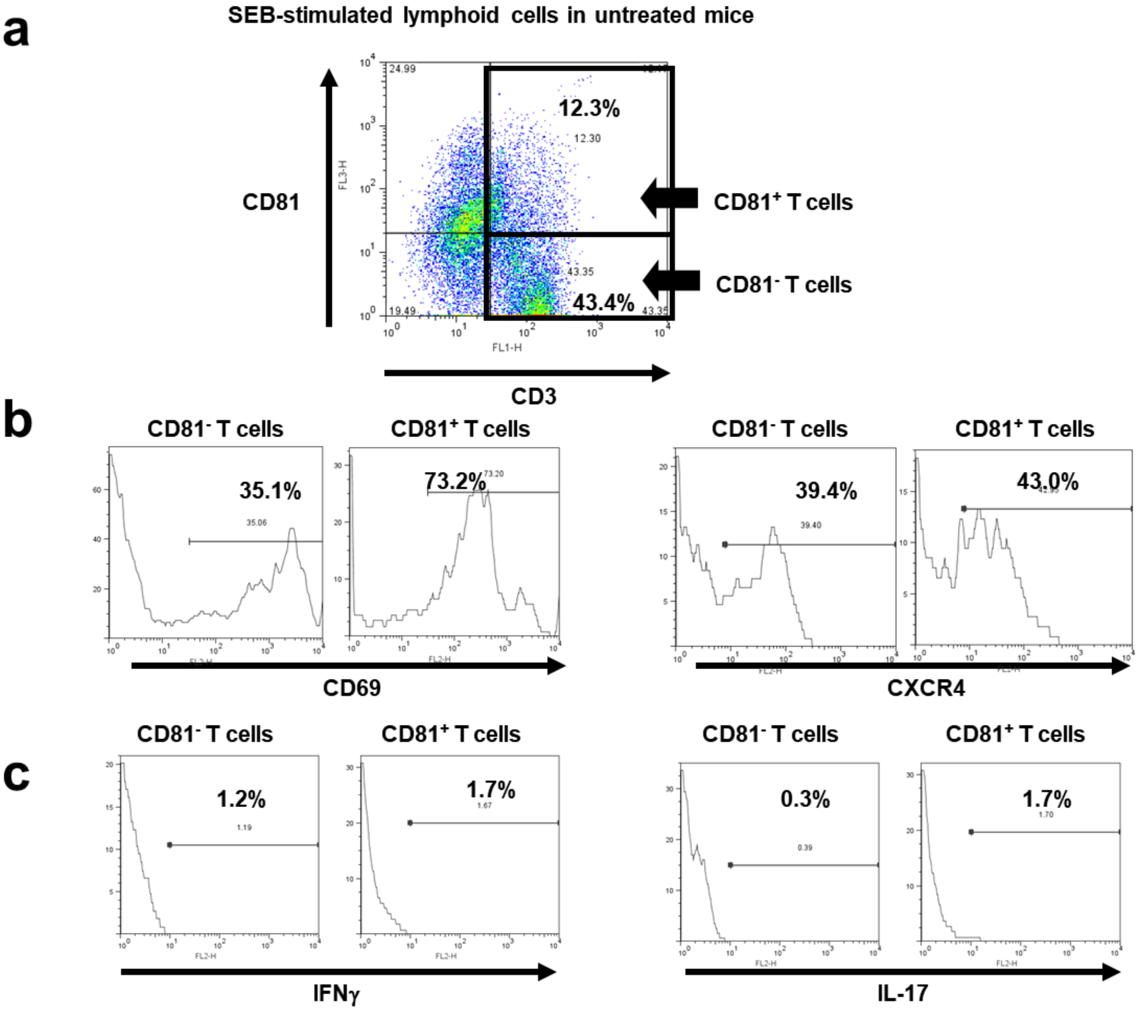

Supplementary Figure 8. CD81$^+$ T cells in spleens of untreated mice are active and migrate, but not cytokine-producing effector T cells. Splenocytes from untreated mice were stimulated with 1 µg/mL SEB for 50 h. The cells were treated with Golgi Stop for 14 h. (a, b) Cell surface molecules (CD81, CD69, and CXCR4) and (c) intracellular cytokines (IFNγ and IL-17) were stained and analysed using the FACSCanto II.

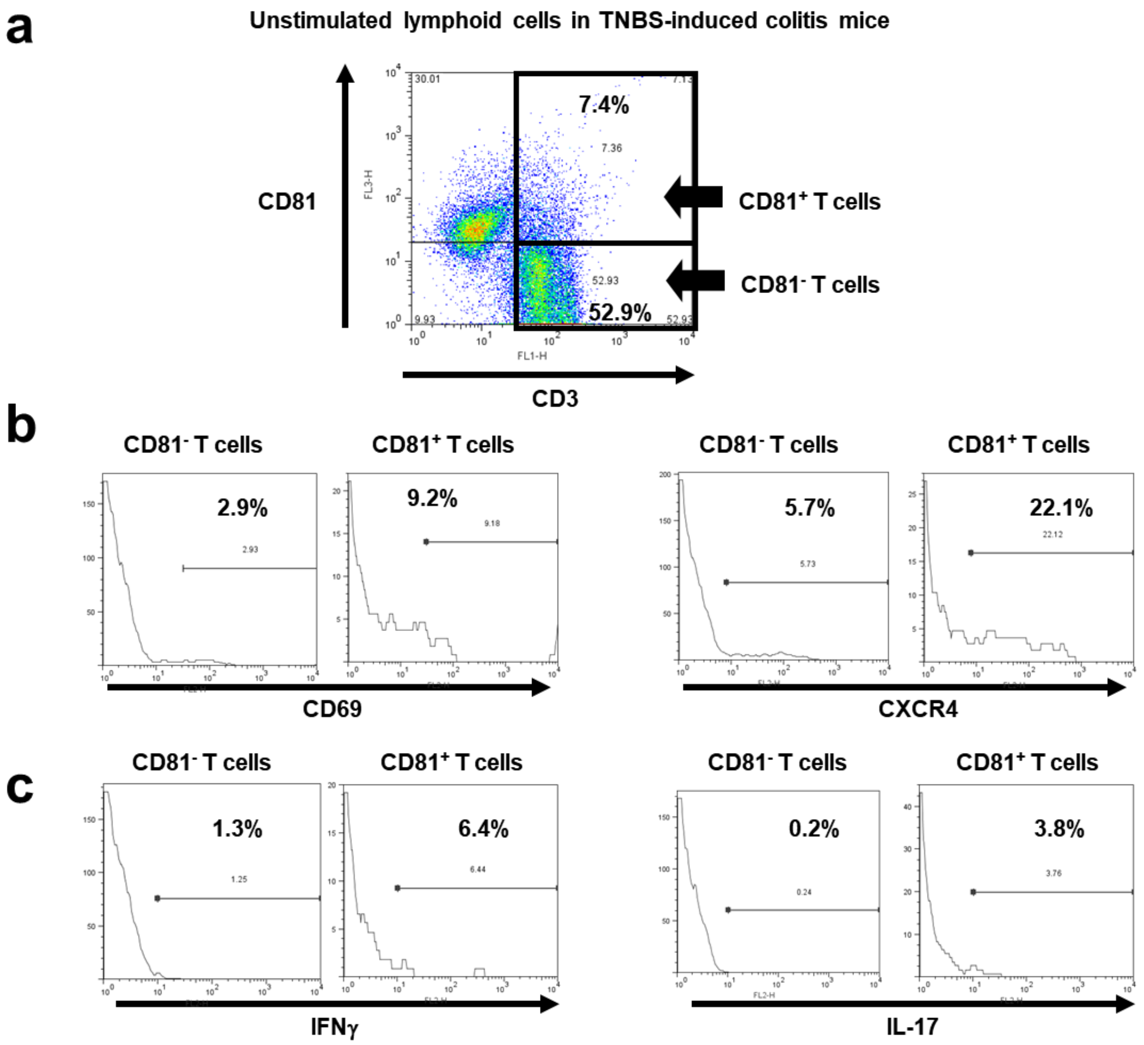

Supplementary Figure 9. CD81$^+$ T cells in spleens from mice with TNBS-induced colitis are active, migrate, and cytokine-producing effector T cells. Splenocytes from mice with TNBS-induced colitis were cultured without stimulation for 50 h. The cells were treated with Golgi Stop for 14 h. (a, b) Cell surface molecules (CD81, CD69, and CXCR4) and (c) intracellular cytokines (IFNγ and IL-17) were stained and analysed using the FACSCanto II.

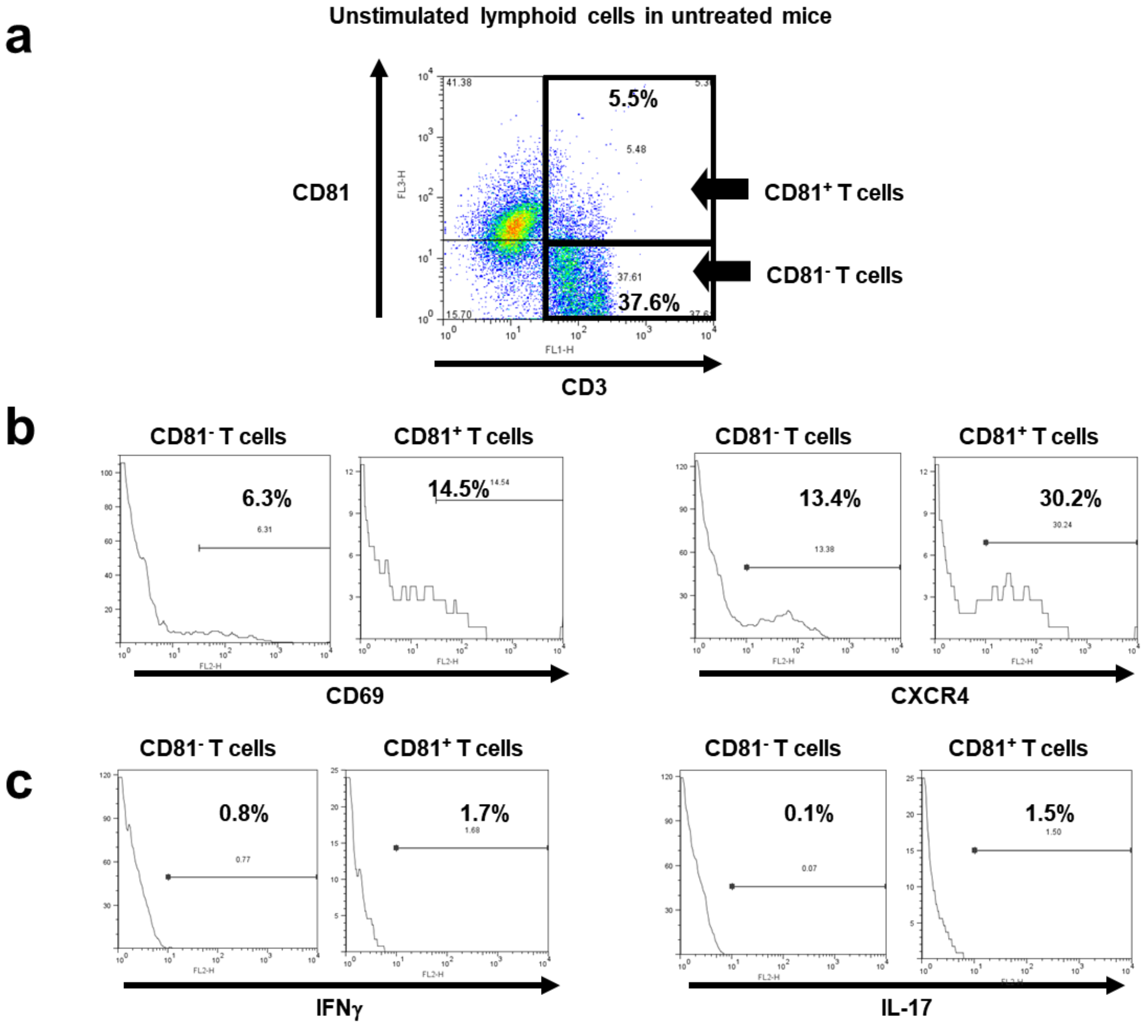

Supplementary Figure 10. CD81^{+} T cells in in spleens of untreated mice are active and migrate, but not cytokine-producing effector T cells. Splenocytes from untreated mice were cultured without stimulation for 50 h. The cells were treated with Golgi Stop for 14 h. (a, b) Cell surface molecules (CD81, CD69, and CXCR4) and (c) intracellular cytokines (IFNγ and IL-17) were analysed using the FACSCanto II.

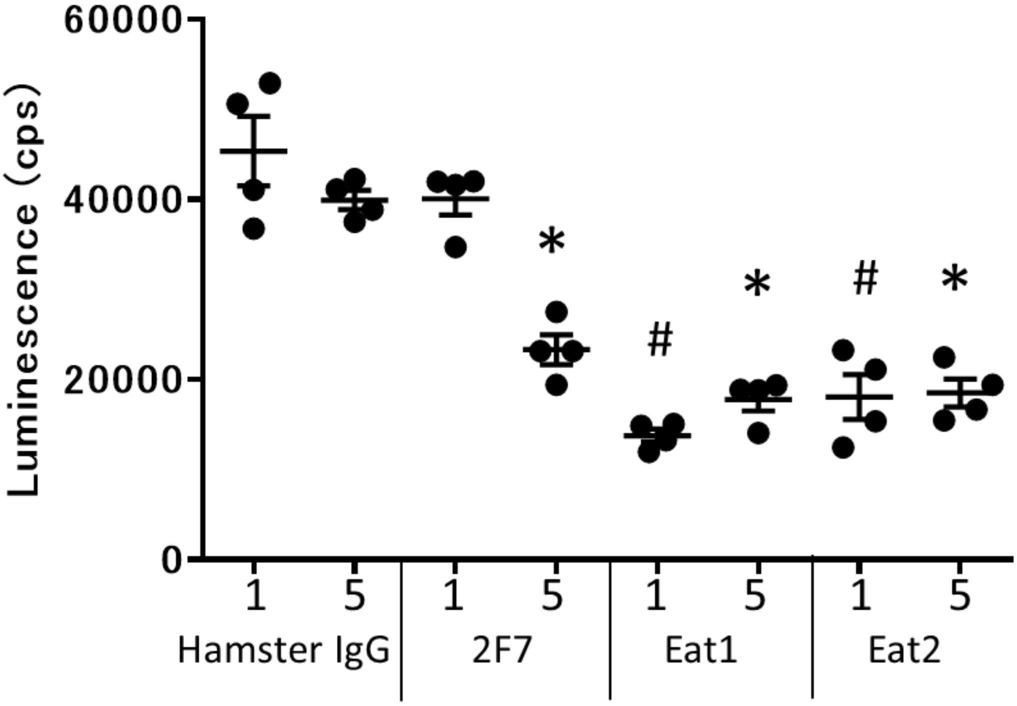

Supplementary Figure 11. Anti-CD81 antibody inhibits EL4IL2 cell migration induced by SDF-1. The mouse T cell line EL-4.IL-2 was incubated with hamster IgG and anti-CD81 antibodies (2F7, Eat1, and Eat2) at 1 or 5 μg/mL for 2 h. Cells were seeded in the upper chamber of a 96-well plate with 5-μm pore transwells with or without 10 ng/mL SDF-1 in the lower chamber for migration assays. The number of cells was counted by ATPlite (n = 4 per group). Data are representative of at least three independent experiments. Statistical significance was determined using the Student's t-test (*p<0.05, 5 μg/mL control IgG vs. anti-CD81 antibody; #p<0.05, 1 μg/mL control IgG vs. anti-CD81 antibody).

a

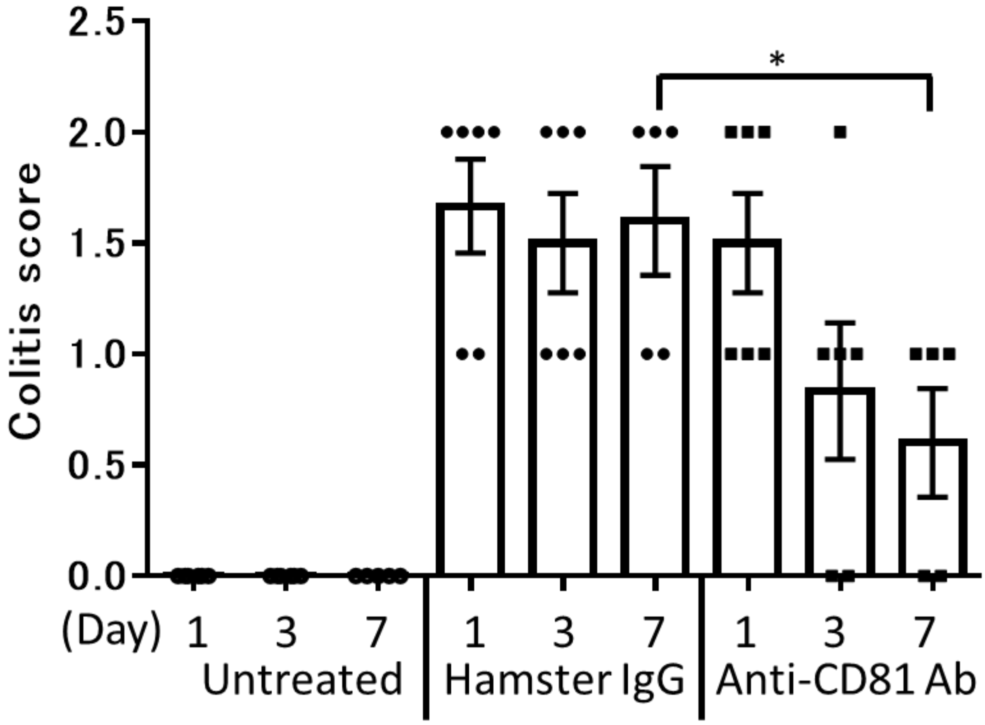
Colitis score
2.5
2.0
1.5
1.0
0.5
0.0
*
(Day) 1 3 7 1 3 7 1 3 7
Untreated
Hamster IgG
Anti-CD81 Ab

b

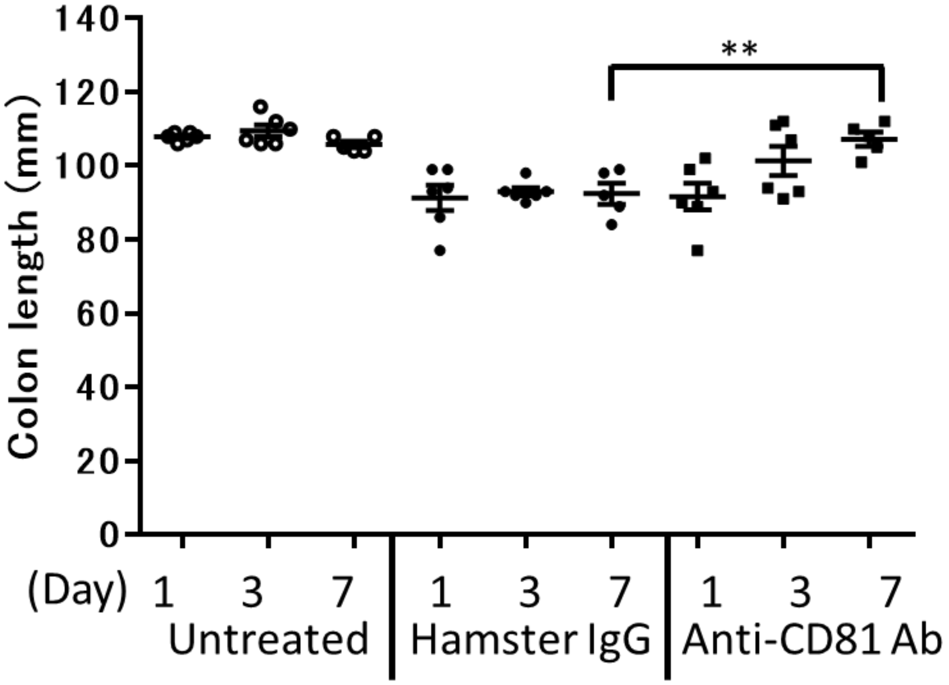
Colon length (mm)
140
120
100
80
60
40
20
0
**
(Day) 1 3 7 1 3 7 1 3 7
Untreated
Hamster IgG
Anti-CD81 Ab

Supplementary Figure 12. Time course of anti-CD81 antibody treatment of TNBS-induced colitis. TNBS was administered intrarectally on day -5. Colitic mice were injected intraperitoneally with hamster IgG at 0.5 mg/mouse and the anti-CD81 antibody (Eat2) at 0.5 mg/mouse on day 0 (n = 6, per group for days 1 and 3, n = 5, per group for day 7). Data are representative of two independent experiments. (a) Colitis scores were calculated on days 1, 3, and 7. Statistical significance was determined using Wilcoxon's test (*$p < 0.05$, hamster IgG vs anti-CD81 antibody) (b) Colons were removed and their lengths measured on days 1, 3, and 7. Statistical significance was determined using the Student's t-test (**$p < 0.01$, hamster IgG vs anti-CD81 antibody)

Supplementary Table 1 Development ratio of TNBS-induced colitis

	TNBS-treated mice (N1)	Excluded mice (N2) and reasons for exclusion			Included mice (N3)	Ratio of colitis development (N3/N1, %)
		Colitis score	Body weight	Dead mice		
**Figure 2**	**60**	**16**	**3**	**16**	**25**	**41.7**
**Figure 3**	**70**	**16**	**10**	**20**	**24**	**34.3**
**Figure 4**	**90**	**19**	**6**	**25**	**40**	**44.4**
**Supplementary Figure 5**	**82**	**18**	**5**	**19**	**40**	**48.8**

Figure 2: Colitis score was 2 and body weight change ranged from 0% to -30%

Figure 3: Colitis score was 1 or 2 and body weight change ranged from 0% to -20%

Figure 4 and Supplementary Figure 5: Colitis score was 1 or 2 and body weight change was less than 0% to -30%.
